# Supplementary material for: Optimising personal continuity for older patients in general practice: a cluster randomised stepped wedge pragmatic trial
Source: BMJ Open. 2024 May 21;14(5):e078169. doi: 10.1136/bmjopen-2023-078169 (PMC11110588; doi:10.1136/bmjopen-2023-078169)
Supplement: Supplementary data [file bmjopen-2023-078169supp001.pdf]

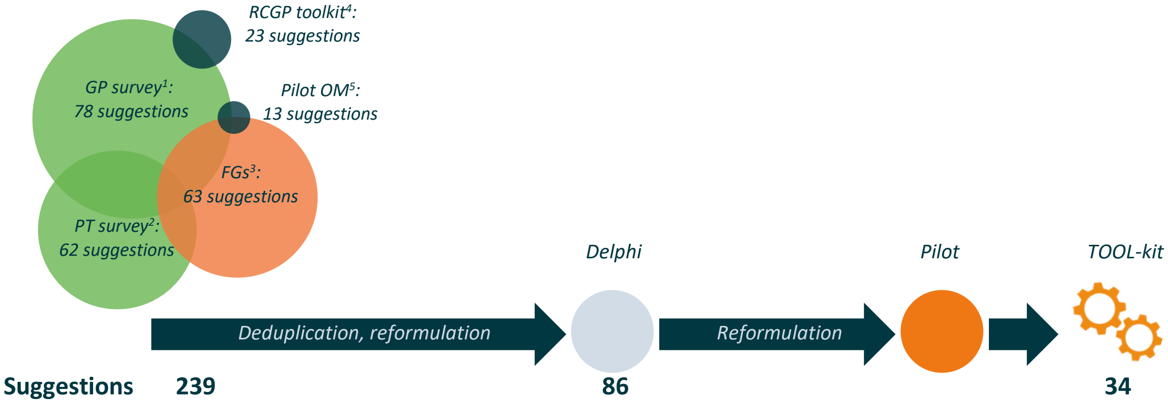

Supplementary figure 1. Development of TOOL-kit.

GP= general practitioner, PT=patient, FG= focus group, RCGP= royal college of general practitioners.

[1] Survey among 249 GPs. *[Groot et al, BJGP open 2023]*

[2] Survey among 1499 patients. *[Groot et al, BJGP open 2023]*

[3] Four focus groups, two with 17 GPs and one with 7 patients and one with 6 practice assistants/nurses.

[4] Available from: https://www.networks.nhs.uk/nhs-networks/releasing-capacity-in-general-practice/messageboard/5-productive-work-flows/750537798/996838860/rcgp-continuity-of-care-toolkit-141119-pdf

[5] Detailed in **Supplementary material S3**
