# Supplementary material for: Optimising personal continuity for older patients in general practice: a cluster randomised stepped wedge pragmatic trial
Source: BMJ Open. 2024 May 21;14(5):e078169. doi: 10.1136/bmjopen-2023-078169 (PMC11110588; doi:10.1136/bmjopen-2023-078169)
Supplement: Supplementary data [file bmjopen-2023-078169supp002.pdf]

Supplementary material S2. Contents of TOOL-kit

Table of contents

Appendix 2. the TOOL-kit 1
1. The TOOL-kit (English) 2
How does the TOOL-kit work? 3
Step 1. Run the Practice Scan 5
Step 2. Select strategies for improvement 6
Step 3. Drafting a practice improvement plan 7
2. de TOOL-kit (Dutch) 41
Hoe werkt de TOOL-kit? 42
Stap 1. De praktijkscan 44
Stap 2. Selectie van verbetersuggesties 45
Stap 3. Praktijkdoelen opstellen 45

Groot L, *et al. BMJ Open* 2024; 14:e078169. doi: 10.1136/bmjopen-2023-078169

1. TOOL-kit (English)

Dear General practitioner,

Welcome to TOOL-kit. This application allows you to create a Practice Improvement Plan for improving personal continuity in the elderly.

During a nationwide reassessment of the core values and tasks of General practitioner (GP) care in the Netherlands, it became clear that GPs still consider continuity of care to be one of the core values of the GP profession. However, due to changes in society and in healthcare, this core value is coming under increasing pressure.

To improve continuity, we have – after extensive consultation with patients, GPs, practice assistants and practice nurses – developed TOOL-kit (op**T**imising pers**O**nal c**O**ntinuity for o**L**der patients). This instrument helps you to improve the practice improvement plan for improving personal continuity for elderly patients in your practice.

With the TOOL-kit, you can create your own Practice Improvement Plan that is tailored to your GP practice.

Groot L, *et al. BMJ Open* 2024; 14:e078169. doi: 10.1136/bmjopen-2023-078169

How does TOOL-kit work?

TOOL-kit contains the following components:

- Step 1. Run GP Practice Scan
- Step 2. Select improvement suggestions
- Step 3. Set practice goals
- Step 4. Implement practice improvement plan in your practice

Step 1. Run Practice Scan

The Practice Scan consists of a questionnaire with 34 yes/no questions that identifies opportunities for improving practice improvement plan for improving personal continuity in your GP practice.

1. *Start filling in the Practice Scan at **arrow 1.***

   - You decide how to go through this practice scan. For example, you can do this by yourself, but you can also choose to do this with your colleagues during a team meeting.
   - Completing the GP Practice Scan takes about 10 minutes.

Step 2. Select improvement suggestions

The GP Practice Scan will first give you several suggestions for improving practice improvement plan for improving personal continuity in your GP practice. You then select the improvement suggestions that seem most suitable for your practice.

2. *Put the page with the Practice Scan next to the page with the suggestions. put a cross in the column under **arrow 2** for each item where you answered 'No' on the practice scan*
3. *Select a suggestions by putting a cross in the column under **arrow 3.***

   - There is no minimum or maximum number of improvement suggestions that you can select.
   - You decide how to select the suggested improvements. For example, you can do this by yourself, but you can also choose to do this with your colleagues during a team meeting.
   - Selecting the improvement suggestions will take approximately 10 minutes.

Step 3. Drafting a practice improvement plan

Each improvement suggestion is linked to a practice goal and to a proposed approach and activities. The proposed approach provides a guideline for how you can achieve this practical goal. By following the approach, you set a practical goal according to the SMART* methodology. The total of all practice goals set this way, is your practice improvement plan.

*S: specific; M: measurable; A: achievable; R: realistic; T: time-bound

4. *For each suggestion, there is a step-by-step guide available to support you in formulating a practice goal. The guides for each suggestion can be found on the page number under **arrow 4.** Take note of these suggested approaches and formulate a practice goal for each suggestion selected in step 2.*

- You decide whether and how you use the suggested approach and activities and how you set the practice goals. For example, you can do this by yourself, but you can also choose to do this with your colleagues during a team meeting.
- Setting the practice goals will take 15-30 minutes, depending on the number of improvement suggestions selected.

Step 4. Implement the practice improvement plan
You now implement the practice improvement plan as formulated in step 3 according to its contents. Your GP practice, family medicine in general, and the characteristics and wishes of your patient population are all subject to change. We therefore advise you to periodically reflect on practice improvement plan for improving personal continuity in your practice and to evaluate or re-evaluate the state of affairs.

1

Step 1. Run the Practice Scan

		Yes	No
1	Are all patients in your practice registered in the name of a permanent GP?		
2	Do all GPS in a permanent position, including salaried GPs and permanent locum GPs, have patients registered to their name?		
3	When you open a patient record in your GP EPR system, is it immediately clear which GP is linked to that patient?		
4	Do Practice assistants schedule appointments with the patient's regular GP by default?		
5	Do Practice assistants schedule consultations for a complex problem, such as a chronic illness or psychological symptoms, with the patient's regular GP by default?		
6	Do Practice assistants schedule follow-up consultations by telephone with the patient's regular GP by default?		
7	Do Practice assistants schedule follow-up consultations for a single problem with the GP that the patient visited for the first consultation by default?		
8	Are home visits for housebound patients routinely conducted by one or two regular GPs ?		
9	Are repeat prescriptions only written by the patient's regular GP, if possible?		
10	Are laboratory results assessed and discussed with the patient by the same GP who requested the test?		
11	Are all GPs who work less than 0.8 FTE in patient care coupled with another GP?		
12	Do patients with complex problems have a second contact person besides their regular GP?		
13	Do part-time working GPs who are coupled with another GP (duo-doctors) regularly discuss their patients?		
14	Do all GPs offer a face-to-face meeting to newly registered patients aged 65 years and over?		
15	Does every GP periodically call patients with complex problems to ask how they are doing?		
16	Does the patient's regular GP contact patients when they return home after being admitted to hospital for a major treatment or event, by default?		
17	Do you use the GP EPR system to identify patients with low personal continuity?		
18	Does your practice website provide up-to-date information on staff, and on working hours, working days and planned absences of healthcare providers?		
19	Does your practice periodically send out a newsletter to patients about any changes in staff and in working hours and working days of healthcare providers?		
20	Can patients request an electronic consultation (e-consultation)?		
21	Can patients make appointments for consultation with the healthcare provider of their choice via the internet?		
22	Do GPs offer telephone consultation to their own patients on working days when they do not hold regular consultation hours (non-consultation days)?		
23	Does your practice have a call-back list for telephone consultations that patients can subscribe to?		
24	Is the standard consultation time 15 minutes?		
25	Is there a policy for the availability of GPs outside of office hours if other GPs from your own practice or those from the local GP out-of-hours centre wish to consult with them?		
26	Is there a policy for the availability of GPs for terminally ill patients outside of office hours?		
27	Do all permanent GPs in the practice hold consultation hours on at least 3 different days per week?		
28	Does your practice rarely employ locum GPs or substitute GPs?		
29	Is there no overlap of the holidays of two GPs who share a patient population?		
30	Do locum GPs routinely write a handover report?		
31	Are diagnostic considerations and personal reflections systematically recorded in the GP EPR system?		
32	Is it standard practice for the practice nurse to inform the patient's regular GP when they learn that a patient has experienced a life event, and vice versa?		
33	Do the GPs periodically perform a diabetes check-up in their patients with diabetes mellitus themselves?		
34	In the case of patients with complex problems, do their records contain a clear summary of the most important data, such as medication, main diagnoses, current problem areas and information on their contact persons?		

2 3 4

Step 2. Select strategies for improvement

☐	Every patient is on the personal list of a GP	☐	Page 7
☐	All GPs in the practice have patients registered on their personal list	☐	Page 8
☐	Patient records in the EPR system display a pop-up message with the name of the patient's regular GP	☐	Page 9
☐	Practice assistants preferably schedule appointments with the patient's regular GP	☐	Page 10
☐	Practice assistants preferably schedule appointments for patients with complex problems with the patient's regular GP	☐	Page 11
☐	Follow-up telephone consultations for one illness episode are conducted by the patient's regular GP	☐	Page 12
☐	One problem, one GP	☐	Page 13
☐	Home visits for housebound patients are conducted by one or two regular GPs	☐	Page 14
☐	Repeat prescriptions are preferably issued by the patient's regular GP	☐	Page 15
☐	Laboratory results are assessed and discussed with the patient by the requesting GP	☐	Page 16
☐	Part-time GPs work together in duos	☐	Page 17
☐	Patients with complex problems have two regular GPs	☐	Page 18
☐	Duo-doctors have regular consultation meetings	☐	Page 19
☐	All GPs in the practice offer face-to-face meetings to newly registered patients aged 65 years and over	☐	Page 20
☐	All GPs in the practice have regular telephone contact with patients with complex problems	☐	Page 21
☐	The patient's regular GP contacts patients when they return home after hospital admission	☐	Page 22
☐	Structured and regular identification of patients with low continuity of care	☐	Page 23
☐	Patients are regularly informed of organisational changes in the practice via the practice website	☐	Page 24
☐	Patients are regularly informed of organisational changes in the practice via a newsletter	☐	Page 25
☐	Patients can request e-consultation	☐	Page 26
☐	Patients can make appointments online with the healthcare provider of their choice	☐	Page 27
☐	GPs offer telephone consultation to their own patients on non-consultation days	☐	Page 28
☐	A call-back list for telephone consultations is managed by the practice	☐	Page 29
☐	The standard consultation time is 15 minutes	☐	Page 30
☐	GPs are available outside of office hours for inter collegial consultations on complex patients	☐	Page 31
☐	GPs are available for terminally ill patients outside of office hours	☐	Page 32
☐	Every permanent GP offers consultation hours at least 3 days a week	☐	Page 33
☐	Minimise the number of locums	☐	Page 34
☐	GPs who share the care of a patient population do not go on holiday at the same time	☐	Page 35
☐	Locum GPs write a handover report by default	☐	Page 36
☐	GPs record their medical considerations and personal reflections systematically in the EPR system	☐	Page 37
☐	Practice nurses and GPs inform each other of patients' life events	☐	Page 38
☐	The patient's regular GP performs one quarterly diabetes check-up per year in their diabetic patients by default	☐	Page 39
☐	The records of patients with complex problems contain a medical summary	☐	Page 40

Groot L, *et al. BMJ Open* 2024; 14:e078169. doi: 10.1136/bmjopen-2023-078169

Step 3. Drafting a practice improvement plan

Practice Goal 1: Every patient is on the personal list of a GP

All patients in the practice are registered in the name of a regular GP and not in the name of the partnership. This way, patients, Practice assistants, practice nurses and GPs know who the primary contact person for an individual patient is. This also facilitates building a trusting relationship between the patient and one healthcare provider.

Suggested approach and activities:

- ☐ Determine which staff members you would like to be involved in reaching the practice goal. Record this and inform the staff involved.
- ☐ Record when you would like this practice goal to be achieved.
- ☐ Determine how patients will be registered in the name of their regular GP.
    - *For example, you can register all new patients or office patients only. Or you can identify patients in your GP Electronic Patient Record (EPR) system using specific search terms, such as patient number, zip code or surname.*
    - *If you have limited time or staff to register patients, then please consider prioritising certain patient groups, such as patients aged 65 years and over.*
- ☐ Determine how many patients will be assigned to each GP. In doing so, please take into account any differences in full-time equivalent (FTE) between GPs.
- ☐ Determine who will be registering the patients in the name of their regular GP and set a start date.
- ☐ Discuss with your fellow GPs when, to what extent and how patients will have a say in who will become their regular GP.
- ☐ In the GP EPR system, link the name of patients to their assigned GP.
    - *If you do not know how to link a GP to a patient, please contact the helpdesk of your GP EPR system.*
- ☐ Inform patients of the name of their regular GP, for example, by letter, via email or verbally during office visits.
- ☐ Decide how and when you will monitor the outcome of this improvement suggestion.
    - *For example, on pre-selected dates, you can assess the percentage of patients who have been registered in the name of their regular GP and then compare these to the predetermined target values.*

Practice Goal 2: All GPs in the practice have patients registered on their personal list

If not all GPs in a permanent position have patients registered to their name, there could be an imbalance in their workloads. This makes it more difficult for patients to make an appointment with their 'own' GP. By distributing patients evenly among all permanent GPs, it is easier for the GP to get to know their own patients and follow up on them.

Suggested approach and activities:

This approach requires that patients are listed to a GP's name in the GP EPR system.

- ☐ Determine which staff members you would like to be involved in reaching the practice goal. Record this and inform the staff involved.
- ☐ Record when you would like this practice goal to be achieved.
- ☐ Discuss with your fellow GPs which GPs will have patients registered to their name.
    - *Please bear in mind that having your own patients – and therefore more responsibility – can lead to resistance among locum and salaried GPs. Practice owners, on the other hand, may experience resistance because they have to relinquish 'their' patients.*
- ☐ Determine how many patients will be assigned per GP FTE.
- ☐ Determine how patients will be registered in the name of all permanent GPs.
    - *For example, you can register all new patients or office patients only. Or you can identify patients in your GP EPR system using specific search terms, such as patient number, zip code or surname.*
    - *If you have limited time or staff to register patients, then please consider prioritising certain patient groups, such as patients aged 65 years and over.*
- ☐ Determine yourself or discuss with the staff involved who will be registering patients in name of the permanent GPs and when.
- ☐ Discuss with your fellow GPs when, to what extent and how patients will have a say in who will become their regular GP.
- ☐ In the GP EPR system, link the name of the GP to the patients that have been registered to their name.
    - *If you do not know how to link a GP to a patient, please contact the helpdesk of your GP EPR system.*
- ☐ Inform patients of the name of their regular GP.
- ☐ Decide how and when you will monitor the outcome of this improvement suggestion.
    - *For example, you can assess the percentage of permanent GPs with patients registered to their name by FTE ratio over time.*

Groot L, *et al. BMJ Open* 2024; 14:e078169. doi: 10.1136/bmjopen-2023-078169

Practice Goal 3: Patient records in the EPR system display a pop-up message with the name of the patient's regular GP

By using a pop-up message, Practice assistants, GPs and other healthcare providers are made aware of the name of the patient's regular GP. This way, an appointment can be scheduled with this GP to promote personal continuity.

Suggested approach and activities:

This approach requires that patients are listed to a GP's name in the GP EPR system.

- ☐ Determine which staff members you would like to be involved in reaching the practice goal. Record this and inform the staff involved.
- ☐ Determine the number of patient records that should display the pop-up message to achieve the practice goal.
  - o *For example, all patient records or a selection of records should contain the pop-up message.*
- ☐ Record when you would like this practice goal to be achieved.
- ☐ If necessary, please check first how pop-up messages can be implemented in your GP EPR system.
  - o *If you do not know how to add pop-up messages to patient records, please contact the helpdesk of your GP EPR system.*
- ☐ Determine to which patient records you want to add a pop-up message.
  - o *For example, you can add a pop-up message to the records of all new patients or only to those of office patients. Or you can identify patient records in your GP EPR system using specific search terms, such as patient number, zip code or surname.*
  - o *If you have limited time or staff to add pop-up messages, then please consider prioritising certain patient groups, such as patients aged 65 years and over.*
- ☐ Discuss or decide who will add the pop-up messages to the selected patient records in the GP EPR system and when.
- ☐ Educate Practice assistants on the usefulness of the pop-up message and instruct them to always open the patient record in the GP EPR when scheduling an appointment.
- ☐ Consider how to ensure that the staff involved (continue to) act on the pop-up message.
- ☐ Is your staff experiencing 'pop-up fatigue'? Determine which pop-ups are of most value and remove the unnecessary ones.
- ☐ Decide how and when you will monitor the outcome of this improvement suggestion.
  - o *For example, you can assess the percentage of patient records that have a pop-up message showing the name of the patient's regular GP.*

Practice Goal 4: Practice assistants should preferably schedule appointments with the patient's regular GP

When scheduling appointments, Practice assistants give priority to scheduling an appointment with the patient's regular GP. As a result, the patient sees their regular healthcare provider more often, which contributes to personal continuity.

Suggested approach and activities:

This approach requires that patients are listed to a GP's name in the GP EPR system.

- ☐ Determine which staff members you would like to be involved in reaching the practice goal. Record this and inform the staff involved.
- ☐ Determine the extent to which Practice assistants should schedule patients with the patient's regular GP to achieve the practice goal.
- ☐ Record when you would like this practice goal to be achieved.
- ☐ Discuss with your fellow GPs how to deal with urgent consultations.
- ☐ Instruct Practice assistants to always check the GP EPR system first when scheduling appointments to see who the patient's regular GP is. The assistants then try to schedule the appointment with this GP and not with another GP who is available earlier unless there are medical reasons for doing so.
    - o *When upon opening a patient record in the GP EPR system it is not immediately clear who the patient's regular GP is, please consider adding a pop-up message with the name of the patient's regular GP to the patient records in the system.*
- ☐ Consider how to ensure that the assistants (continue to) act in accordance with your instructions.
    - o *For example, you can repeat the instructions periodically.*
    - o *Please evaluate the way assistants schedule appointments in a separate meeting.*
- ☐ Consider informing patients they may have to wait longer for an appointment.
- ☐ Decide how and when you will monitor the outcome of this improvement suggestion.

    Examples:
    - o *You can have the assistants or GPs keep a diary for 1 week per month, in which they will keep track of the number of appointments they have scheduled and how often they have succeeded in scheduling these with the patient's regular GP.*
    - o *The GPs check the appointment schedule at the end of the day during a work week and count the number of consultations with their own patients. This number is divided by the total number of (their) consultations on that day.*

Groot L, *et al. BMJ Open* 2024; 14:e078169. doi: 10.1136/bmjopen-2023-078169

Practice Goal 5: Practice assistants should preferably schedule appointments for patients with complex problems with the patient's regular GP

Patients with complex problems benefit more from practice improvement plan for improving personal continuity. When assistants give priority to scheduling appointments with the patient's regular GP, the patient will see their regular healthcare provider more often. This contributes personal continuity.

Suggested approach and activities:

This approach requires that patients are listed to a GP's name in the GP EPR system.

- ☐ Determine which staff members you would like to be involved in reaching the practice goal. Record this and inform the staff involved.
- ☐ Determine the extent to which the assistants should schedule patients with complex problems with the patient's regular GP to achieve the practice goal.
- ☐ Record when you would like this practice goal to be achieved.
- ☐ Determine the definition of a 'complex problem'.
  - *Complexity is determined by various factors, such as chronic psychological symptoms, multimorbidity, an adverse social situation, care-avoiding behaviour or a combination of these factors.*
  - *To identify patients with complex problems, you can use specific ICPC codes or combinations of multiple ICPC codes.*
- ☐ Determine how assistants are able to identify patients with complex problems.
- ☐ Discuss with your fellow GPs how to deal with urgent consultations of complex patients.
- ☐ Instruct Practice assistants to ask the patient whether they have a complex problem when scheduling appointments. In that case, the assistants check the GP EPR system first to see who the patient's regular GP is. They then try to schedule the appointment with this GP and not with another GP who is available earlier unless there are medical reasons for doing so.
  - *If the patient wishes to make an appointment for a non-complex problem, they can do so with any GP.*
  - *When upon opening a patient record in the GP EPR system it is not immediately clear who the patient's regular GP is, please consider adding a pop-up message with the name of the patient's regular GP to the patient records in the system.*
- ☐ Consider how to ensure that the assistants (continue to) act in accordance with your instructions.
  - *For example, you can repeat the instructions periodically.*
  - *Please evaluate the way assistants schedule appointments in a separate meeting.*
- ☐ Consider informing patients they may have to wait longer for an appointment.
- ☐ Decide how and when you will monitor the outcome of this improvement suggestion.

  Examples:
  - *You can have the assistants or GPs keep a diary for 1 week per month, in which they will keep track of the number of appointments they have scheduled and how often they have succeeded in scheduling these with the patient's regular GP.*
  - *The GPs check the appointment schedule at the end of the day during a work week and count the number of consultations with their own complex patients. This number is divided by the total number of (their) consultations with complex patients on that day. This number is monitored.*

Groot L, et al. BMJ Open 2024; 14:e078169. doi: 10.1136/bmjopen-2023-078169

Practice Goal 6: Follow-up telephone consultations for one illness episode are conducted by the patient's regular GP

By scheduling follow-up consultations with the patient's regular GP, the patient will see their regular healthcare provider more frequently. It also allows the GP to follow up on the previous consultation. As a result, the GP and the patient get to know each other better, which contributes to personal continuity.

Suggested approach and activities:

This approach requires that patients are listed to a GP's name in the GP EPR system.

- ☐ Determine which staff members you would like to be involved in reaching the practice goal. Record this and inform the staff involved.
- ☐ Determine the extent to which the assistants should schedule follow-up telephone consultations for one illness episode with the patient's regular GP to achieve the practice goal.
- ☐ Record when you would like this practice goal to be achieved.
- ☐ Discuss with all staff involved how to deal with urgent follow-up telephone consultations.
- ☐ Instruct Practice assistants to always check the GP EPR system first when scheduling follow-up phone calls to see who the patient's regular GP is. The assistants then try to schedule a follow-up consultation by telephone with this GP and not with another GP who is available earlier unless there are medical reasons for doing so.
  - *When upon opening a patient record in the GP EPR system it is not immediately clear who the patient's regular GP is, please consider adding a pop-up message with the name of the patient's regular GP to the patient records in the system.*
- ☐ Consider how to ensure that the assistants (continue to) act in accordance with your instructions.
  - *For example, you can repeat the instructions periodically.*
  - *Please evaluate the way assistants schedule appointments in a separate meeting.*
- ☐ Consider informing patients they may have to wait longer for an appointment.
- ☐ Decide how and when you will monitor the outcome of this improvement suggestion.

  Examples:
  - *You can have the assistants or GPs keep a diary for 1 week per month, in which they will keep track of the number of follow-up telephone consultations they have scheduled and how often they have succeeded in scheduling these with the patient's regular GP.*
  - *The GPs check the appointment schedule at the end of the day during a work week and count the number of follow-up telephone consultations with their own patients. This number is divided by the total number of (their) follow-up telephone consultations on that day. This number is monitored.*

Groot L, *et al. BMJ Open* 2024; 14:e078169. doi: 10.1136/bmjopen-2023-078169

Practice Goal 7: One problem, one GP

In order to have patients return to the same GP as much as possible, consultations for one disease episode are preferably scheduled with the same GP. As a result, the patient can build a relationship with this GP, which contributes to practice improvement plan for improving personal continuity.

Suggested approach and activities:

- ☐ Determine which staff members you would like to be involved in reaching the practice goal. Record this and inform the staff involved.
- ☐ Determine the extent to which the assistants should schedule an appointment for one problem with one GP to achieve the practice goal.
- ☐ Record when you would like this practice goal to be achieved.
- ☐ Discuss with your fellow GPs in which case it is better for a patient to see their regular GP instead of the GP they visited for the first consultation.
- ☐ Discuss with all staff involved how to deal with urgent follow-up consultations.
- ☐ Instruct Practice assistants to ask when scheduling an appointment whether the patient has already seen a GP for the current problem and, if so, which GP that was. If the patient does not know, the assistant should check the GP EPR system first to see if the patient has recently seen a GP for this problem.
  - *When upon opening a patient record in the GP EPR system it is not immediately clear who the patient's regular GP is, please consider adding a pop-up message with the name of the patient's regular GP to the patient records in the system.*
- ☐ Consider how to ensure that the assistants (continue to) act in accordance with your instructions.
  - *For example, you can repeat the instructions periodically.*
  - *Please evaluate the way assistants schedule appointments in a separate meeting.*
  - *Patients may have to wait longer for an appointment. This also requires support and adaptation of the assistants.*
- ☐ Decide how and when you will monitor the outcome of this improvement suggestion.
  Examples:
  - *You can have the assistants or GPs keep a diary for 1 week per month, in which they will keep track of the number of follow-up appointments they have scheduled and how often they have succeeded in scheduling these with the GP who saw the patient for the first consultation.*
  - *The GPs check the appointment schedule at the end of the day during a work week and count the number of follow-up consultations with patients they also saw for the first consultation. This number is divided by the total number of (their) follow-up consultations on that day. This number is monitored.*

Practice Goal 8: Home visits for housebound patients are conducted by one or two regular GPs

Please ensure that patient records in the GP EPR system display a pop-up message for patients who are temporarily or permanently unable to come to the practice. This pop-up message lists the names of the regular GPs that will make house calls for this patient. As a result, the patient sees only one or two regular GPs, which promotes practice improvement plan for improving personal continuity.

<u>Suggested approach and activities</u>:

- ☐ Determine which staff members you would like to be involved in reaching the practice goal. Record this and inform the staff involved.
- ☐ Determine the extent to which home visits for housebound patients should be conducted by one or two regular GPs to achieve the practice goal.
- ☐ Record when you would like this practice goal to be achieved.
- ☐ Discuss with your team of GPs which patients should be considered as 'housebound' and how they can be identified in the GP EPR system.
- ☐ Determine which GPs will conduct home visits to housebound patients. This could be the patient's regular GP.
- ☐ Determine how the GP EPR system will display which regular GPs are allowed to make house calls for these patients.
    - o *For example, you can register all housebound patients in the name of their regular GPs or add a pop-up message to their record in the GP EPR system with the names of the GPs authorised to conduct home visits.*
- ☐ Determine who will be identifying these patients and how and when this should be registered in the GP EPR system.
- ☐ Instruct Practice assistants to open patient record in the GP EPR system when scheduling appointments for home visits and to act in accordance with the EPR system's instructions for dealing with housebound patients.
- ☐ Consider how to ensure that the assistants (continue to) act in accordance with your instructions.
    - o *For example, you can repeat the instructions periodically.*
    - o *Please evaluate the way assistants schedule appointments in a separate meeting.*
    - o *Patients may have to wait longer for an appointment. This also requires support and adaptation of the assistants.*
- ☐ Decide how and when you will monitor the outcome of this improvement suggestion.

    Examples:
    - o *You can have the assistants or GPs keep a diary for 1 week per month, in which they will keep track of the number of housebound patients for whom they have scheduled a home visit with the patient's regular GP.*
    - o *The GPs check the appointment schedule at the end of the day during a work week and count the number of home visits for their own housebound patients. This number is divided by the total number of home visits for housebound patients on that day. This number is monitored.*

Practice Goal 9: Repeat prescriptions are preferably issued by the patient's regular GP

When repeat prescriptions are issued by the GP who knows the patient, this can reduce the number of medication errors and the number of unjustified repeat prescriptions (e.g. due to excessive prescription of benzodiazepines or opiates).

Suggested approach and activities:

- ☐ Determine which staff members you would like to be involved in reaching the practice goal. Record this and inform the staff involved.
- ☐ Determine the extent to which repeat prescriptions should be issued by the patient's regular GP to achieve the practice goal.
    - *For example, only repeat prescriptions for one specific drug class (such as benzodiazepines or opiates) should be written by the patient's regular GP.*
- ☐ Record when you would like this practice goal to be achieved.
- ☐ Discuss with your team of GPs how to facilitate that repeat prescriptions are issued by the patient's regular GP by default.
- ☐ Set a policy for how urgent repeat prescriptions are processed when the patient's regular GP is not present.
- ☐ Set a deadline for processing non-urgent repeat prescriptions.
- ☐ Inform the entire team of GPs about the new procedure for writing repeat prescriptions.
- ☐ Consider informing patients they may have to wait longer for a non-urgent repeat prescription and that they need to ask for a repeat prescription in time.
- ☐ Decide how and when you will monitor the outcome of this improvement suggestion.
    - *For example, you can assess the percentage of repeat prescriptions issued by the patient's regular GP in relation to the total number of repeat prescriptions for that patient over a specified period of time.*

Groot L, *et al. BMJ Open* 2024; 14:e078169. doi: 10.1136/bmjopen-2023-078169

Practice Goal 10: Laboratory results are assessed and discussed with the patient by the requesting GP

The requesting GP knows the reason for requesting certain diagnostic tests and can therefore better interpret the results in the context of the patient's current problem. By discussing the laboratory results with the patient, the requesting GP can provide personalised care and improve the doctor-patient relationship.

Suggested approach and activities:

- ☐ Determine which staff members you would like to be involved in reaching the practice goal. Record this and inform the staff involved.
- ☐ Determine the extent to which laboratory results should be handled by the requesting GP to achieve the practice goal.
- ☐ Record when you would like this practice goal to be achieved.
- ☐ Discuss with your team of GPs the need for adapting the practice's procedures so that the GP who requests a laboratory test also discusses them with the patient and, if necessary, schedules a follow-up appointment with the patient.
- ☐ Decide together on the exceptions, for example, in the case of emergency diagnostic testing or abnormal laboratory results.
- ☐ Set a deadline for processing non-urgent laboratory results.
  - *If there are GPs in your practice who work less than 3 days a week or if there are many different locum GPs, setting a deadline for the assessment of laboratory results is of even more importance.*
- ☐ Consider informing patients they may have to wait longer for non-urgent laboratory results.
- ☐ Consider how to ensure that the assistants (continue to) act in accordance with your instructions.

  Examples:
  - *You can repeat the instructions periodically.*
  - *You can plan a meeting with the assistants in which you evaluate the way laboratory results are dealt with.*
- ☐ Decide how and when you will monitor the outcome of this improvement suggestion.
  - *For example, you can – at the end of each day, for a week – assess the number of laboratory results that have been dealt with by the requesting GP in the GP EPR system. This number is then divided by the total number of laboratory results that have been dealt with that day.*

Practice Goal 11: Part-time GPs work together in duos

Patients of GPs who work part-time are assigned a second regular GP. As a result, these patients will see fewer different healthcare providers and are able to build a better relationship with their GPs.

Suggested approach and activities:

- ☐ Determine which staff members you would like to be involved in reaching the practice goal. Record this and inform the staff involved.
- ☐ Record when you would like this practice goal to be achieved.
- ☐ Make a list of GPs working part-time (< 0.8 FTE) who can form a duo with which of the other GPs working full-time or part-time.
    - *Please ensure that these duo-doctors can guarantee one of them is available for patient care at least 4 days a week.*
- ☐ Discuss your proposal with your fellow GPs and record which GPs will be paired together.
- ☐ Determine who will be registering the duo-doctors in the GP EPR system and how and when they will do this.
    - *If you have limited time or staff to register all patients in the name of the duo-doctors in time, then please consider introducing these changes one at a time or prioritising certain patient groups, such as patients aged 65 years and over.*
- ☐ Instruct Practice assistants to check the GP EPR system to see who the patient's duo-doctors are, if any, when scheduling appointments. The assistants should preferably schedule appointments with one of the two regular duo-doctors.
    - *Please consider adding a pop-up message with the names of the patient's regular duo-doctors to the patient records in the GP EPR system.*
- ☐ Decide, possibly together with your colleagues, when and to what extent patients will have a say in who will become their second contact person.
    - *Please take into account that some patients prefer to visit only one GP and no others.*
- ☐ Inform these patients that they have a second contact person and how they can have a say in the choice of who their GPs are.
- ☐ Consider how to ensure that the assistants (continue to) act in accordance with your instructions.
    - *For example, you can repeat the instructions periodically.*
    - *Please evaluate the way assistants schedule appointments in a separate meeting.*
- ☐ Decide how and when you will monitor the outcome of this improvement suggestion.
    - *For example, you can make a list with the names of all permanent GPs, their FTEs and whether they are working as duo-doctors.*

Practice Goal 12: Patients with complex problems have two regular GPs

Patients with complex problems benefit more from practice improvement plan for improving personal continuity. When these patients have a maximum of two regular GPs, they will see fewer different healthcare providers and are able to build a better relationship with their GPs.

Suggested approach and activities:

- ☐ Determine which staff members you would like to be involved in reaching the practice goal. Record this and inform the staff involved.
- ☐ Record when you would like this practice goal to be achieved.
- ☐ Determine the definition of a ‘complex problem’.
  - *Complexity is determined by various factors, such as chronic psychological symptoms, multimorbidity, an adverse social situation, care-avoiding behaviour or a combination of these factors.*
  - *To identify patients with complex problems, you can use specific ICPC codes or combinations of multiple ICPC codes.*
- ☐ Determine who will identify patients with complex problems in the GP EPR system and how and when they will do this.
- ☐ Together with your fellow GPs, appoint a second GP for each patient with a complex problem, in addition to the regular GP. This second GP is the patient’s second contact person.
  - *Please ensure that these two GPs can guarantee one of them is available for patient care at least 4 days a week.*
- ☐ Determine who will register the second contact person for these patients in the GP EPR system and how and when they will do this.
- ☐ Instruct Practice assistants to check the GP EPR system to see who the patient’s regular GPs are, if any, when scheduling appointments. The assistants should preferably schedule appointments with one of the two regular GPs.
  - *Please consider adding a pop-up message with the names of the patient’s regular GPs to the patient records in the GP EPR system.*
- ☐ Decide, possibly together with your colleagues, when and to what extent patients will have a say in who will become their second contact person.
  - *Please take into account that some patients prefer to visit only one GP and no others.*
- ☐ Inform these patients that they have a second contact person and how they can have a say in the choice of who their GPs are.
- ☐ Consider how to ensure that the assistants (continue to) act in accordance with your instructions.
  - *For example, you can repeat the instructions periodically.*
  - *Please evaluate the way assistants schedule appointments in a separate meeting.*
- ☐ Decide how and when you will monitor the outcome of this improvement suggestion.

  Examples:
  - *You can make a list with the names of patients with complex problems and indicate for each patient which two GPs are their regular contact persons.*
  - *In your GP EPR system, you can run a search query to assess the number of patients with complex problems who have been assigned a second regular GP.*

Practice Goal 13: Part-time GPs working in duos have regular consultation meetings

Part-time GPs who share patients have regular consultation meetings. This ensures coordinated treatment of patients, as well as discussion of their individual observations and considerations. Together, this personal continuity.

Suggested approach and activities:

This approach assumes that your practice employs Part-time GPs working in duos (duo-doctors).

- ☐ Determine which staff members you would like to be involved in reaching the practice goal. Record this and inform the staff involved.
- ☐ Determine the frequency and duration of these consultation meetings.
  - *Depending on the specific working days of the duo-doctors, it may be difficult to schedule these consultation meetings.*
- ☐ Record when you would like this practice goal to be achieved.
- ☐ Determine the content of the consultation meetings.
  - *Please take into account that having additional consultation meetings means there is less time for other tasks, such as patient consultation hours.*
- ☐ Decide whether duo-doctors may be disturbed during their consultation meetings for consultation by telephone and what the policy is for any emergencies arising during their meetings.
- ☐ Decide how and when you will monitor the outcome of this improvement suggestion.

  Examples:
  - *You can assess the number of consultation meetings between duo-doctors per month.*
  - *You can assess the number of actions taken on the basis of the consultation meetings.*

Groot L, *et al. BMJ Open* 2024; 14:e078169. doi: 10.1136/bmjopen-2023-078169

Practice Goal 14: All GPs in the practice offer face-to-face meetings to newly registered patients aged 65 years and over

Patients appreciate it when their GP takes initiative, and an introductory interview can facilitate building a doctor-patient relationship of trust.

Suggested approach and activities:

- ☐ Determine which staff members you would like to be involved in reaching the practice goal. Record this and inform the staff involved.
- ☐ Record when you would like this practice goal to be achieved.
- ☐ Determine which patients aged 65 years and over your practice will invite for an introductory meeting.
    - *For example, you can invite all new patients aged 65 and over, all new patients aged 65 and over registered over the past 3 months, or patients aged 65 and over with chronic diseases only.*
    - *If your practice has many new patients aged 65 and over, the time for regular consultations may decrease and the waiting time for patients may increase.*
- ☐ Determine who will invite patients to the introductory meetings and how and when they will do this.
    - *Please consider directing patients to reliable online information on introductory meetings with GPs when inviting them.*
- ☐ Decide how and when you will monitor the outcome of this improvement suggestion.
    - *For example, you can – for a year – assess the percentage of new patients aged 65 and over who are offered an introductory interview. This number is then divided by the total number of patients aged 65 and over.*

Practice Goal 15: All GPs in the practice have regular telephone contact with patients with complex problems

Patients appreciate it when their GP takes initiative. By talking with them regularly, the GP keeps track of patients with complex problems. practice improvement plan for improving personal continuity is promoted by an increased number of contacts and the GP's proactive attitude.

Suggested approach and activities:

- ☐ Determine which staff members you would like to be involved in reaching the practice goal. Record this and inform the staff involved.
- ☐ Record when you would like this practice goal to be achieved.
- ☐ Determine the definition of a 'complex problem'.
    - *Complexity is determined by various factors, such as chronic psychological symptoms, multimorbidity, an adverse social situation, care-avoiding behaviour or a combination of these factors.*
    - *To identify patients with complex problems, you can use specific ICPC codes or combinations of multiple ICPC codes.*
    - *If many of your patients are identified as being 'complex', this practice goal may become too time-consuming. To make the goal more achievable, please consider using a narrower definition of a 'complex problem'.*
- ☐ Determine who will identify patients with complex problems in the GP EPR system and how and when they will do this.
- ☐ Make a list of patients with complex problems and their regular GP.
- ☐ Discuss with your fellow GPs how telephone consultations with these patients will be organised. Determine how frequently, for how long and when telephone consultations should be conducted. The topics of these calls should be discussed as well.
- ☐ Decide how and when you will monitor the outcome of this improvement suggestion.

    Examples:
    - *You can assess the number of GPs in the practice who have a call list of complex patients.*
    - *You can assess the number of telephone contacts initiated by the GP for complex patients per time interval.*

Practice Goal 16: The patient's regular GP contacts patients when they return home after hospital admission for a treatment or event that had a profound impact on the patient

Most patients feel the need to talk with their regular GP when they experience a major event. They believe that their GP knows their general medical condition and personal situation well. By contacting the patient after hospitalisation for a major treatment or event, the GP shows interest. This also strengthens the doctor-patient relationship.

Suggested approach and activities:

- ☐ Determine which staff members you would like to be involved in reaching the practice goal. Record this and inform the staff involved.
- ☐ Record when you would like this practice goal to be achieved.
- ☐ Together with your fellow GPs, determine the definition of a 'major event'.
  - *The definition of a 'major event' may differ per patient. You may consider compiling a list of events that are always major, such as the diagnosis of a serious illness.*
- ☐ Discuss with your fellow GPs how patients will be contacted after learning they have been discharged from hospital after a major event.
- ☐ Determine the time frame within which patients should have been contacted.
  - *When you experience problems with communication from the hospital, it may take longer before patients can be contacted after discharge.*
- ☐ Instruct the Practice assistants who handle mail that discharge notifications for patients who were admitted for a major event should be made known to the patient's regular GP.
- ☐ Decide how and when you will monitor the outcome of this improvement suggestion.
  Examples:
  - *You can set a policy for contacting patients after hospital admission for a major event.*
  - *You can have GPs keep track of how frequently they have planned to contact a patient after hospital admission for a major event on their own initiative over a specified period of time.*
  - *In your GP EPR system, you can run a search query to assess the percentage of patients who were contacted by their regular GP after hospital admission for a major event.*

Groot L, *et al. BMJ Open* 2024; 14:e078169. doi: 10.1136/bmjopen-2023-078169

Practice Goal 17: Structured and regular identification of patients with low continuity of care

A GP EPR system search is conducted periodically to identify patients with low continuity of care. This way, GPs can monitor which patients have lower practice improvement plan for improving personal continuity and decide which continuity-promoting actions should be taken.

Suggested approach and activities:

- ☐ Determine which staff members you would like to be involved in reaching the practice goal. Record this and inform the staff involved.
- ☐ Determine who will extract patient data from the GP EPR system and calculate the UPC index.
- ☐ Determine for which patient population you want to calculate the UPC index (e.g. patients aged 65 years and over or patients with multimorbidity).
- ☐ In the GP EPR system, conduct a search for the specified patient population and extract the consultation data for these patients. Assess the number of consultations they have had over the past 2 years and with which GP, and who their regular GP is.
  - *If you do not know how to run a search query, please contact the helpdesk of your GP EPR system.*
- ☐ Calculate the UPC index by entering the data on the patients and consultations in the UPC index calculator from the University of Bristol.
  - *The UPC index is calculated by dividing the patient's total number of visits with their regular GP by the total number of visits of that patient with any GP over a specified period of time. The UPC index scale runs from 0 to 1, with 0 indicating 'minimal continuity of care' and 1 indicating 'perfect continuity of care'.*
- ☐ Compile a list of patients with the lowest UPC index.
  - *For example, you can include all patients with a UPC index < 0.5 or those with a UPC index in the lowest 10%.*
- ☐ Discuss the list with your fellow GPs and determine which actions should be taken and when.
  Examples:
  - *You can have a Practice assistant invite all patients with low continuity or care for an office visit.*
  - *All patients with low continuity of care are called by their regular GP.*
  - *All patients with low continuity of care are registered in the name of their regular GP.*
- ☐ Decide who and when will repeat the GP EPR system search and any subsequent actions.
- ☐ Decide how and when you will monitor the outcome of this improvement suggestion.
  Examples:
  - *Is there a list of patients with a low UPC index and the name of the GP who will contact them? Yes/No*
  - *You can monitor the UPC index of all patients over the course of a year.*

Practice Goal 18: Patients are regularly informed of the current staff members and of working hours and working days of healthcare providers via the practice website

The practice website contains up-to-date information on which healthcare providers work at the practice and on which days. This way, patients can decide for themselves whether they are willing to wait to see their regular GP, which makes them less likely to go to a locum GP.

Suggested approach and activities:

- ☐ Determine which staff members you would like to be involved in reaching the practice goal. Record this and inform the staff involved.
- ☐ Determine who will be responsible for updating the practice website and when and how frequently this should be updated. In addition, determine which current information about the healthcare providers should be listed on the website.
  - o *For example, the website contains information on current working days, consultation hours, dates of planned absences, and staff departures and arrivals.*
  - o *If there is an intermittent staff change, for example due to departure, illness or pregnancy, please adjust the website as soon as possible.*
- ☐ Consider how to ensure that the practice website is updated every 3 months.
- ☐ Decide how and when you will monitor the outcome of this improvement suggestion.
  - o *For example, you can check periodically whether the information on the practice website is up to date.*

Practice Goal 19: Patients are regularly informed of changes in staff and in working hours and working days of healthcare providers via a newsletter

The practice sends out a periodic newsletter, either digitally or by mail, which informs patients of any changes in the working hours or working days of healthcare providers and of any staff changes. This way, patients can decide for themselves whether they are willing to wait to see their regular GP, which makes them less likely to go to a locum GP.

Suggested approach and activities:

- ☐ Determine which staff members you would like to be involved in reaching the practice goal. Record this and inform the staff involved.
- ☐ Decide who will send the newsletter and when and how frequently. In addition, decide how the newsletter will be sent: by email or regular mail.
- ☐ Discuss which information is to be included in the newsletter and record this.
    - *For example, the newsletter contains information on current working days, consultation hours, notifications of dates of planned absences, and staff departures and arrivals.*
    - *If there is an intermittent staff change, for example due to departure, illness or pregnancy, please send out an extra newsletter.*
- ☐ Offer patients the possibility to subscribe or unsubscribe to this newsletter (opt-in or opt-out procedure).
- ☐ Decide how and when you will monitor the outcome of this improvement suggestion.
  Examples:
    - *Is there a newsletter template for current practice information? Yes/No*
    - *You can assess the number of times a newsletter has been sent over a specified period of time.*

Practice Goal 20: Patients can request e-consultations

The practice will offer e-consultation to patients, which will increase the accessibility of the patient's regular GP, and vice versa. This makes it easier for patients to ask for help from a GP who knows them.

Suggested approach and activities:

- ☐ Determine which staff members you would like to be involved in reaching the practice goal. Record this and inform the staff involved.
- ☐ Record when you would like this practice goal to be achieved.
- ☐ Contact a software vendor to ask about the possibilities of implementing e-consultation.
    - *Please make sure the software you want to buy complies with your country's regulatory standards, such as the international standard for information security (ISO/IEC 27001) and the EU's General Data Protection Regulation.*
    - *Depending on your ICT infrastructure and other software packages, there may be additional costs for organising e-consultation.*
- ☐ Discuss the organisation, application and set-up of e-consultation with your fellow GPs and the Practice assistants.
- ☐ Determine who (and how and when) will inform patients and explain how they can request e-consultation.
- ☐ Decide how and when you will monitor the outcome of this improvement suggestion.
    - *For example, can patients request e-consultation? Yes/No*

Practice Goal 21: Patients can make appointments online with the healthcare provider of their choice

Patients can make appointments online with their regular healthcare provider. This way, the practice is more accessible for patients than if they had to call, and it is also easier for patients to make an appointment with a healthcare provider they know.

Suggested approach and activities:

- ☐ Determine which staff members you would like to be involved in reaching the practice goal. Record this and inform the staff involved.
- ☐ Record when you would like this practice goal to be achieved.
- ☐ Contact a software vendor to ask about the possibilities of implementing online appointment scheduling.
    - *Depending on your ICT infrastructure and other software packages, there may be additional costs for organising online appointment scheduling.*
- ☐ Determine who (and how and when) will inform patients and explain how they can make appointments online.
- ☐ Decide how and when you will monitor the outcome of this improvement suggestion.
  Examples:
    - *Can patients make appointments online? Yes/No*
    - *You can assess the percentage of appointments that have been made online per time unit.*

Practice Goal 22: GPs offer telephone consultation to their own patients on non-consultation days

There is a time slot for telephone consultation for a GP's own patients on non-consultation days. This improves the GP's accessibility for their own patients, and it also makes it easier for patients to have personal contact with their regular GP.

<u>Suggested approach and activities</u>:

- ☐ Determine which staff members you would like to be involved in reaching the practice goal. Record this and inform the staff involved.
- ☐ Determine the extent to which GPs should hold telephone consultation hours on non-consultation days to achieve the practice goal.
- ☐ Record when you would like this practice goal to be achieved.
- ☐ Discuss with your fellow GPs on which non-consultation days – these are preferably fixed days per week – they will offer telephone consultation hours.
    - *Please bear in mind that scheduling more time for patient care means there is less time for other tasks.*
- ☐ Determine who will introduce the new telephone consultation hours and when.
- ☐ Instruct Practice assistants to only schedule the GP's own patients for the telephone consultation hours.
- ☐ Decide how and when you will monitor the outcome of this improvement suggestion.
  Examples:
    - *You can assess the percentage of non-consultation days on which a time slot has been newly allocated to telephone consultation hours per GP.*
    - *You can keep a file (e.g. in Excel) with the names of the GPs and their availability for patient care on their non-consultation days.*

Practice Goal 23: A call-back list for telephone consultations is managed by the practice

Instead of offering telephone consultation hours where patients call in and are put on hold, they can register for telephone consultation at a specific time. If possible, patients are called back at this time, which improves the GP's accessibility. It also makes it easier for patients to have personal contact with their regular GP.

Suggested approach and activities:

- ☐ Determine which staff members you would like to be involved in reaching the practice goal. Record this and inform the staff involved.
- ☐ Consider how many patients can be called per hour.
- ☐ Determine the time frame within which patients will be called back.
- ☐ Determine how patients can subscribe to the call-back list and consider how this will be communicated to them.
    - *If you would like patients to be able to register for telephone consultation online, please contact the software vendor of your patient portal.*
- ☐ Consider how call-back appointments will be recorded in the GP EPR system.
- ☐ Determine when you will start the call-back list.
- ☐ Instruct Practice assistants to work with the call-back list.
- ☐ Decide how and when you will monitor the outcome of this improvement suggestion.
    - *For example, is there a call-back list which patients can subscribe to? Yes/No*

Groot L, *et al. BMJ Open* 2024; 14:e078169. doi: 10.1136/bmjopen-2023-078169

Practice Goal 24: The standard consultation time is 15 minutes

Pilot studies have shown that having 15-minute consultations lead to fewer referrals and fewer hospital admissions than shorter consultations. By spending more time with the patient, a calm atmosphere is created in the consulting room, patient satisfaction is increased, and the doctor-patient relationship is improved. This promotes practice improvement plan for improving personal continuity.

Suggested approach and activities:

- ☐ Determine which staff members you would like to be involved in reaching the practice goal. Record this and inform the staff involved.
- ☐ Identify the organisational implications of a longer standard consultation time.
  - *Determine whether a longer consultation time affects other agreements concerning consultations, e.g. patients are now allowed to discuss two problems instead of just one.*
  - *Practice assistants may experience more pressure when scheduling appointments because there are fewer time slots per GP FTE available.*
  - *Consider delegating tasks or consultations to a practice nurse or Practice assistant to increase GPs' availability for (complex) patient care.*
- ☐ Discuss with your GP team how to make this practice goal financially feasible.
  - *Consider delegating tasks or consultations to a practice nurse or Practice assistant in order to maintain a higher number of billable consultations.*
- ☐ Discuss with your GP team how to implement the extended standard consultation time in your practice and when this should be done.

  Examples:
  - *You can implement the longer standard consultation time incrementally per GP or for all GPs at once.*
  - *You can implement the longer standard consultation time selectively or partially during fixed times of the day or for specific target groups (e.g. patients aged 65 years and over).*
- ☐ Determine how to inform patients that the standard consultation time has been increased.
  - *Please emphasise both the advantages of a longer consultation time (more time for the patient, fewer referrals, greater satisfaction) and the disadvantages (patients may have to wait longer for an appointment).*
- ☐ Decide how and when you will monitor the outcome of this improvement suggestion.
  - *For example, is the standard consultation time 15 minutes? Yes/No*

Practice Goal 25: GPs are available outside of office hours for inter-collegial consultations on complex patients

GPs set a practice-wide policy to ensure they are available outside of office hours for consultation by other GPs. This does not only concern GPs from the GP out-of-hours centre but also fellow GPs from your own practice on days when the GP in question is not present. This makes GPs more accessible to answer questions about their patients, which promotes personal continuity.

Suggested approach and activities:

- ☐ Determine which staff members you would like to be involved in reaching the practice goal. Record this and inform the staff involved.
- ☐ Record when you would like this practice goal to be achieved.
- ☐ Discuss with your fellow GPs which GPs can be contacted in case of questions from the GP out-of-hours centre about their own patients, how, when and for what type of questions. In doing so, each individual GP should make clear how and when they can be reached outside of office hours.
- ☐ Determine who will communicate this to the GP out-of-hours centre and to other staff of your practice including locum GPs, and how and when they will do this.
- ☐ Decide how and when you will monitor the outcome of this improvement suggestion.
    - *For example, you can keep a file with the names of the GPs, the way they can be reached in case of questions about patients outside of office hours, and at what times.*

Practice Goal 26: GPs are available for terminally ill patients outside of office hours

GPs set a practice-wide policy for their availability regarding terminally ill patients outside of office hours. This can be either direct doctor-patient contact but also consultation by fellow GPs or the GP out-of-office centre. This makes GPs more accessible to the group of patients who need them most (see research by Schers et al.).

Suggested approach and activities:

- ☐ Determine which staff members you would like to be involved in reaching the practice goal. Record this and inform the staff involved.
- ☐ Record when you would like this practice goal to be achieved.
- ☐ Discuss with your fellow GPs which GPs will be available for their terminally ill patients outside of office hours, how, when and for what type of questions.
    - *If your practice employs duo-doctors or if a patient has a second contact person, please consider involving this second GP in making agreements about availability outside of office hours.*
- ☐ Determine who will communicate this to the GP out-of-hours centre, to other staff of your practice including locum GPs, and how they will do this.
- ☐ Decide how and when you will monitor the outcome of this improvement suggestion.

For example, you can keep a file with the names of the GPs, the way they can be reached outside of office hours regarding terminally ill patients, and at what times

Practice Goal 27: Every permanent GP offers consultation hours at least 3 days a week

Every permanent GP is available for direct patient care on at least 3 different days per week, which improves the GP's accessibility. This makes it easier for patients to have personal contact with their regular GP.

Suggested approach and activities:

- ☐ Determine which staff members you would like to be involved in reaching the practice goal. Record this and inform the staff involved.
- ☐ Record when you would like this practice goal to be achieved.
- ☐ Draw up one or more proposals for a new distribution of working days. Please keep in mind that all GPs should be available for patient care on at least 3 different days per week, for at least one half-day.
- ☐ Plan a meeting with your fellow GPs to discuss your proposal for a new distribution of working days and how other commitments including working hours can be moved around to achieve the practice goal.
- ☐ Determine who will inform patients of any changed working days and how and when they will do this.
- ☐ Decide how and when you will monitor the outcome of this improvement suggestion.

  Examples:
  - *You can assess the number of consultation days per GP per week before and after achieving the practice goal.*
  - *You can keep a file (e.g. in Excel) with the names of all permanent GPs and their working days per week.*

Practice Goal 28: Minimise the number of locums

By minimising the use of locum GPs, patients see the same healthcare providers as often as possible, even when their regular GP is absent. This makes it easier to build a doctor-patient relationship of trust.

Suggested approach and activities:

- ☐ Determine which staff members you would like to be involved in reaching the practice goal. Record this and inform the staff involved.
- ☐ Record when you would like this practice goal to be achieved.
- ☐ Determine the conditions for covering the absence of an employee and discuss these conditions with your colleagues.
  - *For example, a GP's short-term absence (e.g. < 2 weeks) is covered by the practice's own staff. Or: a locum GP is called in only in case of long-term absence of a GP.*
  - *Please take into account that the workload of the other employees may increase.*
  - *Please provide adequate overtime compensation.*
- ☐ Decide how and when you will monitor the outcome of this improvement suggestion.
  Examples:
  - *You can keep a file with the names of the GPs who have been absent in the past year and how their absence was covered by employing locum GPs.*
  - *You can make an overview of the number of locum GPs your practice has employed before and after this intervention.*

Practice Goal 29: GPs who share the care of a patient population do not go on holiday at the same time

When the holidays of GPs sharing a patient population do not overlap, there is always at least one regular GP present who knows the patients from this population. This reduces the risk of discontinuity of care.

Suggested approach and activities:

- ☐ Determine which staff members you would like to be involved in reaching the practice goal. Record this and inform the staff involved.
- ☐ Record when you would like this practice goal to be achieved.
- ☐ Discuss the holiday planning with your fellow GPs in advance. Determine how the planning can be adjusted so that GPs who share a patient population do not go on holiday at the same time. Try to ensure that at least one GP who is familiar with the patient population is present in the practice.
    - o *In larger practices or in practices where many GPs have young families as patients, this practice goal may lead to organisational challenges.*
- ☐ Decide how and when you will monitor the outcome of this improvement suggestion.
    - o *For example, you can keep a file with the names of all GPS, their holidays and their planned absences.*

Practice Goal 30: Locum GPs write a handover report

Locum GPs are asked to write a short handover report. This way, the patient's regular GP is systematically informed of important events within their patient population. As a result, the GP can act proactively towards their patients and guarantee practice improvement plan for improving personal continuity.

Suggested approach and activities:

- ☐ Determine which staff members you would like to be involved in reaching the practice goal. Record this and inform the staff involved.
- ☐ Determine when the goal of locum GPs writing a handover report by default is achieved.
- ☐ Record when you would like this practice goal to be achieved.
- ☐ Decide, possibly together with your colleagues, what matters should be included in the handover (e.g. emergencies, to-dos or life events) and how the report should be stored and handed over.
    - *During a busy shift, it can take a lot of time to write a handover report. Therefore, please provide clear instructions on what should be included in the report as a minimum.*
    - *If desired, please consider that some aspects can be reported on orally.*
- ☐ Decide how and when the locum GP will receive the instructions to write the handover report.
- ☐ Decide how and when you will monitor the outcome of this improvement suggestion.

    Examples:
    - *Is there a folder on the hard drive of the practice's computer server in which all handover reports are stored? Yes/No*
    - *You can assess the percentage of locum shifts for which the locum GP has written a handover report.*

Practice Goal 31: GPs record their medical considerations and personal reflections systematically in the EPR system

By recording their considerations regarding diagnostics and/or therapy during consultations, GPs themselves and locum GPs are better able to take decisive actions during follow-up consultations. This type of 'information continuity' will eliminate any problems due to personal discontinuity.

Suggested approach and activities:

- ☐ Determine which staff members you would like to be involved in reaching the practice goal. Record this and inform the staff involved.
- ☐ Record when you would like this practice goal to be achieved.
- ☐ Discuss with your fellow GPs how you want to record considerations and reflections in the GP EPR system.
    - *For example, in a text field, GPs can record their considerations using if-then situations ('If A remains, then think of B or do C') and their feelings about the consultation ('I did not manage to reassure this patient').*
    - *To save time, you can discuss that considerations and reflections should only be recorded for certain patients or for specific consultations.*
    - *When recording reflections and considerations, please bear in mind that patients can (in)directly have access to their record and can therefore 'read' the GPs' notes.*
- ☐ Decide how and when you will monitor the outcome of this improvement suggestion.
  Examples:
    - *In the GP EPR system, you can run a search query on consultations for which the GP's considerations and reflections have been recorded. Together with your fellow GPs, you can evaluate the number of considerations and reflections that are correct and complete.*
    - *You can – periodically and at random – assess the percentage of consultations for which considerations and reflections have been recorded in the GP EPR system.*

Practice Goal 32: Practice nurses and GPs inform each other of patients' life events

If a patient informs the practice nurse that they have experienced a life event, the practice nurse informs the patient's regular GP, and vice versa.

Suggested approach and activities:

- ☐ Determine which staff members you would like to be involved in reaching the practice goal. Record this and inform the staff involved.
- ☐ Determine the extent to which GPs and practice nurses should inform each other of patients' life events to achieve the practice goal.
- ☐ Record when you would like this practice goal to be achieved.
- ☐ Discuss with your team of GPs and practice nurses which life events require practice nurses to inform the patient's regular GP, and vice versa.
    - o *For example, you can make a list of events for which the GP or practice nurse should always be informed.*
- ☐ Determine who will contact patients when they have experienced a life event and when, or if it is better to tailor the approach to the individual patient.
- ☐ Consider how you can ensure that all GPs and practice nurses (continue to) act in accordance with the agreements made.
    - o *For example, you can repeat the instructions periodically.*
- ☐ Decide how and when you will monitor the outcome of this improvement suggestion.
    Examples:
    - o *Is there a list of patients' life events? Yes/No*
    - o *Have practice nurses been instructed on how to inform the patient's regular GP in case of a life event? Yes/No*
    - o *You can assess the number of life events that have/have not been passed on.*

Groot L, *et al. BMJ Open* 2024; 14:e078169. doi: 10.1136/bmjopen-2023-078169

Practice Goal 33: The patient's regular GP performs one quarterly diabetes check-up per year in their diabetic patients by default

The practice nurse (or nurse practitioner) commonly performs the check-ups of diabetic patients. By seeing their own diabetic patients periodically, GPs remain in personal contact with them.

<u>Suggested approach and activities</u>:

- ☐ Determine which staff members you would like to be involved in reaching the practice goal. Record this and inform the staff involved.
- ☐ Record when you would like this practice goal to be achieved.
- ☐ Determine which diabetic patients are being seen by the practice nurse.
- ☐ Determine for which patients the patient's regular GP will perform one quarterly diabetes check-up.
    - *To save time, please consider having the practice-nurse make a selection of patients, such as patients with multimorbidity or those who avoid medical care.*
- ☐ Determine who will schedule the quarterly check-ups for this patient group, and how and when this will be communicated to the patients.
- ☐ Decide how and when you will monitor the outcome of this improvement suggestion.
    - *For example, you can assess the number of quarterly diabetes check-ups performed by the patient's regular GP per patient per year.*

Practice Goal 34: The records of patients with complex problems contain a medical summary

In the GP EPR system, a summary of relevant medical data is included in the records of all complex patients to allow for a smooth handover. You may determine yourself what a 'complex patient' is, for example, patients aged 65 years and over who have more than two chronic conditions.

Suggested approach and activities:

- ☐ Determine which staff members you would like to be involved in reaching the practice goal. Record this and inform the staff involved.
- ☐ Determine the extent to which the records of complex patients should contain a to achieve the practice goal.
- ☐ Determine, possibly together with your colleagues, the definition of a 'complex problem'.
    - *Complexity is determined by various factors, such as chronic psychological symptoms, multimorbidity, an adverse social situation, care-avoiding behaviour or a combination of these factors.*
    - *To identify patients with complex problems, you can use specific ICPC codes or combinations of multiple ICPC codes.*
- ☐ Determine who will identify patients with complex problems in the GP EPR system and how and when they will do this.
- ☐ Discuss what information the summary should contain, for example, current disease episodes, main diagnoses, medication use, advance care planning and contact details of family member or relatives.
    - *Please be aware of any 'registration fatigue' among your healthcare providers.*
- ☐ Determine where the summary will be placed in the GP EPR system so all staff and healthcare providers know where to find this.
    - *If you are not sure where to add the summary, please contact the helpdesk of your GP EPR system.*
- ☐ Determine when you would like this practice goal to be achieved.
    - *When setting a deadline, please bear in mind that writing a summary takes time, which may be at the expense of patient-related tasks.*
- ☐ Decide how and when you will monitor the outcome of this improvement suggestion.
    - *For example, you can assess the percentage of patients with complex problems for whom the patient record contains a summary.*

Groot L, *et al. BMJ Open* 2024; 14:e078169. doi: 10.1136/bmjopen-2023-078169

2. de TOOL-kit (Dutch)

Beste huisarts,

Welkom bij de TOOL-kit. Met deze interactieve richtlijn kunt u een praktijkverbeterplan opstellen voor de verbetering van persoonlijke continuïteit van zorg bij ouderen.

Tijdens de herijking van de kernwaarden en -taken van de huisartsenzorg (Woudschoten, 2019) bleek dat huisartsen continuïteit van zorg nog steeds beschouwen als één van de kernwaarden van het huisartsenvak. Door veranderingen in de maatschappij en in de zorg komt deze kernwaarde echter steeds meer onder druk te staan.

Om de continuïteit te verbeteren, hebben wij – na uitgebreide raadpleging van patiënten, huisartsen, doktersassistenten en praktijkondersteuners – een instrument ontwikkeld: de TOOL-kit. Deze TOOL-kit is een richtlijn dat u helpt om de persoonlijke continuïteit van zorg bij ouderen in uw praktijk te verbeteren.

Met de TOOL-kit stelt u uw praktijkverbeterplan op. Dit is toegespitst op uw huisartsenpraktijk.

Hoe werkt de TOOL-kit?

De TOOL-kit bestaat uit de volgende stappen:

- Stap 1. Praktijkscan
- Stap 2. Verbetersuggesties selecteren
- Stap 3. Praktijkdoelen opstellen
- Stap 4. Praktijkverbeterplan uitvoeren in uw praktijk

Stap 1. Praktijkscan

De praktijkscan bestaat is een vragenlijst met 34 ja/nee-vragen die mogelijkheden identificeert voor verbetering van de persoonlijke continuïteit van huisartsenzorg in uw praktijk.

1. *Start met het invullen van de praktijkscan bij pijl **1**.*

   - U bepaalt zelf hoe u de praktijkscan doorloopt. U kunt dit bijvoorbeeld alleen doen, maar u kunt er ook voor kiezen om de praktijkscan in een teamoverleg gezamenlijk te doorlopen.

   - Het invullen van de praktijkscan kost ongeveer 10 minuten.

Stap 2. Verbetersuggesties selecteren

De praktijkscan laat u een aantal suggesties zien die de persoonlijke continuïteit van huisartsenzorg in uw praktijk kunnen verbeteren. U mag verbetersuggesties selecteren die u het meest geschikt lijken voor uw praktijk.

2. *Leg nu de pagina met de praktijkscan naast de pagina met de verbetersuggesties. Voor elk item waar u "nee" heeft geantwoord, zet u een kruisje in eerste kolom van de verbetersuggesties bij pijl **2**.*
3. *Selecteer een verbetersuggestie door een kruisje te zetten in de kolom bij pijl **3.***

   - Er is geen minimum of maximum aantal verbetersuggesties dat u mag kiezen.

   - U bepaalt zelf hoe u de verbetersuggesties selecteert. U kunt dit bijvoorbeeld alleen doen, maar u kunt er ook voor kiezen om de verbetersuggesties in een teamoverleg gezamenlijk te doorlopen.

   - De selectie van verbetersuggesties kost ongeveer 10 minuten.

Stap 3. Praktijkverbeterplan opstellen

Elke verbetersuggestie is gekoppeld aan een praktijkdoel en een voorgestelde aanpak en activiteiten. De voorgestelde aanpak geeft een handreiking over hoe u dit praktijkdoel kunt bereiken. Door de aanpak te volgen, stelt u een praktijkdoel op volgens de SMART*-methodiek. Het geheel van alle praktijkdoelen is uw praktijkverbeterplan.
*S: specifiek M: meetbaar A: acceptabel R: realistisch T: tijdgebonden

4. *Voor elke verbetersuggestie is er een stappenplan beschikbaar op de pagina die vermeld staat onder **pijl 4**. Neem de stappen voor elke geselecteerde verbetersuggestie door en stel per geselecteerde verbetersuggestie een praktijkdoel op.*

   - U bepaalt zelf of en hoe u de voorgestelde aanpak en activiteiten gebruikt en hoe u de praktijkdoelen opstelt. U kunt dit bijvoorbeeld alleen doen, maar u kunt er ook voor kiezen om de voorgestelde aanpak en activiteiten in een teamoverleg gezamenlijk te doorlopen.

   - Het opstellen van de praktijkdoelen kost 15-30 minuten, afhankelijk van het aantal gekozen verbetersuggesties.

Stap 4. Praktijkverbeterplan uitvoeren in uw praktijk
U voert nu het praktijkverbeterplan uit zoals dat is opgesteld in stap 3. Zowel uw huisartsenpraktijk, de huisartsenzorg in zijn algemeenheid als de kenmerken en wensen van uw patiëntenpopulatie zijn onderhevig aan veranderingen. Het is daarom goed om periodiek stil te staan bij de persoonlijke continuïteit in uw praktijk en te (her)evalueren wat de stand van zaken is.

1

Stap 1. De praktijkscan

		Ja	Nee
1	Staan alle patiënten in uw praktijk op naam van een vaste huisarts en dus niet op naam van de maatschap?		
2	Hebben alle huisartsen met een vaste aanstelling, inclusief HIDHA's en vaste waarnemers, patiënten op naam?		
3	Ziet u direct welke huisarts gekoppeld is aan een patiënt als u een patiëntendossier opent in het HIS?		
4	Plannen assistenten alle patiënten standaard in bij hun vaste huisarts?		
5	Plannen assistenten een consult voor een complex probleem, zoals een chronische ziekte of psychische klachten, standaard in bij de vaste huisarts?		
6	Plannen assistenten een telefonisch vervolgconsult standaard in bij de vaste huisarts?		
7	Plannen assistenten een vervolgconsult voor één probleem standaard in bij de huisarts bij wie de patiënt de eerste keer ook is geweest?		
8	Worden huisbezoeken bij huisgebonden patiënten standaard gedaan door één (of twee) vaste huisarts(en)?		
9	Worden herhaalrecepten, indien mogelijk, alleen uitgeschreven door de vaste huisarts van een patiënt?		
10	Worden de laboratoriumuitslagen beoordeeld en teruggekoppeld door de huisarts die het onderzoek heeft aangevraagd?		
11	Hebben alle huisartsen die minder dan 0,8 fte aan patiëntenzorg leveren een duopartner?		
12	Hebben patiënten met complexe problemen een tweede vast aanspreekpunt, naast hun vaste huisarts?		
13	Is er regelmatig overleg tussen parttime werkende duopartners over hun patiënten?		
14	Bieden alle huisartsen nieuw ingeschreven 65+-patiënten een kennismakingsgesprek aan?		
15	Belt iedere huisarts periodiek patiënten met complexe problemen om te vragen hoe het met hen gaat?		
16	Neemt de vaste huisarts standaard contact op met een patiënt bij thuiskomst na een ziekenhuisopname voor een ingrijpende behandeling of gebeurtenis?		
17	Gebruikt u het HIS om patiënten met een lage continuïteit te identificeren?		
18	Biedt de praktijkwebsite actuele informatie over de medewerkers, over de werktijden en -dagen van zorgverleners en over hun geplande afwezigheid?		
19	Verstuurt de praktijk periodiek een nieuwsbrief aan patiënten over eventuele wijzigingen in personeel en in werktijden en -dagen van zorgverleners?		
20	Hebben patiënten de mogelijkheid om een elektronisch consult (e-consult) aan te vragen?		
21	Hebben patiënten de mogelijkheid om via internet een spreekuurafspraak te maken bij een zorgverlener naar keuze?		
22	Houden huisartsen een telefonisch spreekuur voor eigen patiënten op werkdagen dat ze geen vast spreekuur hebben?		
23	Werkt de praktijk met een terugbellijst voor het telefonisch spreekuur waar patiënten zich op kunnen inschrijven?		
24	Is de standaardconsultduur 15 minuten?		
25	Zijn er afspraken met alle huisartsen over intercollegiale bereikbaarheid buiten reguliere werktijden voor de huisartsenpost en de eigen huisartsenpraktijk?		
26	Zijn er afspraken met alle huisartsen over de bereikbaarheid voor terminaal-palliatieve patiënten buiten kantoortijden?		
27	Houden alle vaste huisartsen in de praktijk spreekuur op minstens 3 verschillende dagen per week?		
28	Schakelt uw praktijk zeer zelden een externe waarnemer of invalkracht in?		
29	Is er geen overlap tussen de vakanties van twee huisartsen die een patiëntenpopulatie delen?		
30	Schrijven waarnemers na waarneming standaard een overdracht?		
31	Worden diagnostische overwegingen en reflecties systematisch genoteerd in het HIS?		
32	Licht de praktijkondersteuner standaard de vaste huisarts in wanneer die praktijkondersteuner verneemt dat een patiënt een levensgebeurtenis heeft meegemaakt, en andersom?		
33	Doen de huisartsen ook periodiek een standaard diabetescontrole bij hun vaste patiënten?		
34	Staat er in het dossier van patiënten met complexe problemen een overzichtelijke samenvatting van de belangrijkste gegevens, zoals medicatie, hoofddiagnosen, actuele probleemgebieden en contactpersonen?		

2 3 4

Stap 2. Selectie van verbetersuggesties

2		3	4
	Zet alle patiënten op naam van een vaste huisarts		blz. 46
	Verdeel de patiënten op naam over alle huisartsen met een vast dienstverband		blz. 47
	Voeg een pop-up toe aan de patiëntendossiers in het HIS met de naam van de vaste huisarts.		blz. 48
	Instrueer de assistenten om, indien mogelijk, alle patiënten standaard in te plannen bij hun vaste huisarts.		blz. 49
	Instrueer de assistenten om, indien mogelijk, patiënten met complexe problemen standaard in te plannen bij hun vaste huisarts.		blz. 50
	Instrueer de assistenten om, indien mogelijk, telefonische vervolgconsulten standaard in te plannen bij vaste huisarts.		blz. 51
	Instrueer de assistenten om, indien mogelijk, patiënten voor een vervolgconsult voor één probleem standaard in te plannen bij de huisarts bij wie de patiënt de eerste keer ook is geweest.		blz. 52
	Leg in het HIS bij huisgebonden patiënten duidelijk vast welke huisarts(en) de huisbezoeken zal/zullen afleggen.		blz. 53
	Herhaalrecepten worden bij voorkeur uitgeschreven door de vaste huisarts van de patiënt.		blz. 54
	De aanvragend huisarts beoordeelt de laboratoriumuitslagen, koppelt deze terug en plant, indien nodig, zelf een vervolgafspraak in voor de patiënt.		blz. 55
	Parttime werkende huisartsen (< 0,8 fte) worden aan elkaar gekoppeld en delen hun patiënten. Deze patiënten krijgen op deze manier twee vaste huisartsen (duodokters).		blz. 56
	Patiënten met complexe problemen krijgen een tweede vast aanspreekpunt, naast hun vaste huisarts.		blz. 57
	Organiseer periodieke overlegmomenten tussen twee duopartners (duodokters).		blz. 58
	Iedere huisarts biedt nieuw ingeschreven 65+-patiënten een kennismakingsgesprek aan		blz. 59
	De huisarts neemt op eigen initiatief periodiek telefonisch contact op met patiënten met complexe problemen.		blz. 60
	De huisarts neemt standaard contact op met een patiënt bij thuiskomst na een ziekenhuisopname voor een ingrijpende behandeling of gebeurtenis		blz. 61
	Gebruik de 'Usual Provider Continuity'-index (UPC-index) in het HIS om patiënten met een lage continuïteit te identificeren en hier acties aan te verbinden.		blz. 62
	Plaats actuele informatie over werktijden, werkdagen en geplande afwezigheid van zorgverleners op de praktijkwebsite.		blz. 63
	Verstuur periodiek een nieuwsbrief over eventuele wijzigingen in personeel en in werktijden en -dagen van zorgverleners, inclusief hun geplande afwezigheid.		blz. 64
	Maak e-consultatie mogelijk		blz. 65
	Maak het mogelijk om via internet spreekuurafspraken te maken bij een zorgverlener naar keuze.		blz. 66
	Voer een kort telefonisch spreekuur in voor eigen patiënten van huisartsen op werkdagen dat ze geen vast spreekuur hebben maar wel werken.		blz. 67
	Voer een terugbellijst voor het telefonisch spreekuur in.		blz. 68
	Verleng de standaardconsultduur zo nodig naar 15 minuten.		blz. 69
	Maak afspraken met collega-huisartsen over hun bereikbaarheid buiten reguliere werktijden voor huisartsen van de huisartsenpost of de eigen huisartsenpraktijk.		blz. 70
	Spreek af in welke mate huisartsen buiten kantoortijden bereikbaar zijn voor terminaal-palliatieve patiënten.		blz. 71
	Iedere vaste huisarts houdt spreekuur op 3 verschillende dagen in de week.		blz. 72
	Verminder het aantal externe waarnemers of invalkrachten door eerst eigen medewerkers de openstaande uren te laten vullen.		blz. 73
	Spreek af om vakanties van huisartsen die een patiëntenpopulatie delen zo min mogelijk te laten overlappen.		blz. 74
	Laat de waarnemer standaard een schriftelijk waarnemingsverslag schrijven na waarneming.		blz. 75
	Noteer overwegingen en reflecties systematisch in het SOEP-systeem van het HIS.		blz. 76
	De praktijkondersteuner en de huisarts lichten elkaar in bij levensgebeurtenissen van patiënten.		blz. 77
	Laat de vaste huisarts van een patiënt met diabetes standaard jaarlijks één kwartaalcontrole doen.		blz. 78
	Schrijf een samenvatting in het HIS-dossier van patiënten met complexe problemen.		blz. 79

Stap 3. Praktijkdoelen opstellen

Praktijkdoel 1: Elke patiënt staat op naam van de vaste huisarts

Alle patiënten in de praktijk staan op naam van een vaste huisarts en niet op naam van de maatschap. Hierdoor is het voor patiënten, assistenten en huisartsen duidelijk wie het vaste aanspreekpunt is voor een patiënt. Dit maakt het ook gemakkelijker om een vertrouwensband op te bouwen met één zorgverlener.

<u>Voorgestelde aanpak en activiteiten</u>:

- ☐ Stel vast welke medewerkers u wilt betrekken bij het praktijkdoel. Registreer en communiceer dit naar de betrokken medewerkers.
- ☐ Leg vast wanneer u dit praktijkdoel bereikt wilt hebben.
- ☐ Bepaal de wijze waarop patiënten op naam worden gezet.
    - *Bijvoorbeeld: zet alle nieuwe patiënten op naam of alleen de spreekuurbezoekers. Of identificeer patiënten via HIS-zoekopdrachten op bepaalde kenmerken, zoals patiëntnummer, postcode of achternaam.*
    - *Hebt u weinig tijd of personeel om patiënten op naam te zetten? Overweeg dan bepaalde patiëntengroepen te prioriteren, zoals 65+-patiënten.*
- ☐ Bepaal hoeveel patiënten worden verdeeld per huisarts. Houd hierbij rekening met eventuele verschillen in fte tussen huisartsen.
- ☐ Bepaal wie de patiënten op naam gaat zetten en stel een startdatum vast.
- ☐ Overleg met uw collega-huisartsen wanneer, in welke mate en op welke wijze patiënten inspraak krijgen in wie hun vaste huisarts wordt.
- ☐ Koppel in uw HIS de naam van de patiënten aan de aan hen toegewezen huisarts.
    - *In alle in Nederland verkrijgbare HIS'en kunt u patiënten op naam zetten. Weet u niet hoe dit moet? Neem dan contact op met de helpdesk van uw HIS.*
- ☐ Communiceer de naam van de vaste huisarts naar de patiënten, bijvoorbeeld per brief, per e-mail of mondeling bij spreekuurbezoek.
- ☐ Besluit hoe en wanneer u de uitkomst van deze verbetersuggestie wilt monitoren.
    - *Bijvoorbeeld: registreer op van tevoren gekozen data het percentage patiënten op naam van een vaste huisarts, bij voorkeur inclusief de vooraf vastgestelde streefwaarden.*

Praktijkdoel 2: Alle vaste huisartsen in uw praktijk hebben patiënten op hun naam staan

Wanneer niet alle vaste huisartsen patiënten op hun naam hebben staan, kan er een scheve werkverdeling ontstaan. Hierdoor is het lastiger voor patiënten om een afspraak te maken bij de 'eigen' huisarts. Door patiënten evenredig te verdelen over alle vaste huisartsen wordt het voor de huisarts eenvoudiger om zijn of haar eigen patiënten te leren kennen en te volgen.

Voorgestelde aanpak en activiteiten:

Voor deze aanpak is het noodzakelijk dat patiënten op naam staan in het HIS.

- ☐ Stel vast welke medewerkers u wilt betrekken bij het praktijkdoel. Registreer en communiceer dit naar de betrokken medewerkers.
- ☐ Leg vast wanneer u dit praktijkdoel bereikt wilt hebben.
- ☐ Bespreek met uw collega-huisartsen welke huisartsen patiënten op naam krijgen.
    - *Houd er rekening mee dat het krijgen van eigen patiënten – en dus van meer verantwoordelijkheid – tot weerstand kan leiden onder waarnemers en HIDHA's. Praktijkhouders daarentegen kunnen weerstand ervaren doordat ze afstand moeten doen van 'hun' patiënten.*
- ☐ Bepaal hoeveel patiënten per huisarts-fte worden verdeeld.
- ☐ Bepaal de wijze waarop alle vaste huisartsen patiënten op naam krijgen.
    - *Bijvoorbeeld: zet alle nieuwe patiënten op naam of alleen de spreekuurbezoekers. Of identificeer patiënten via HIS-zoekopdrachten op bepaalde kenmerken, zoals patiëntnummer, postcode of achternaam.*
    - *Hebt u weinig tijd of personeel om patiënten op naam te zetten? Overweeg dan bepaalde patiëntengroepen te prioriteren, zoals 65+-patiënten.*
- ☐ Bepaal of overleg met de betrokken medewerkers wie, wanneer de patiënten op naam van de vaste huisartsen zet.
- ☐ Overleg met uw collega-huisartsen wanneer, in welke mate en op welke wijze patiënten inspraak krijgen in wie hun vaste huisarts wordt.
- ☐ Koppel in uw HIS de naam van de huisarts aan de aan hen toegewezen patiënten.
    - *In alle in Nederland verkrijgbare HIS'en kunt u patiënten op naam zetten. Weet u niet hoe dit moet? Neem dan contact op met de helpdesk van uw HIS.*
- ☐ Communiceer de naam van de vaste huisarts naar de patiënten.
- ☐ Besluit hoe en wanneer u de uitkomst van deze verbetersuggestie wilt monitoren.
    - *Bijvoorbeeld: registreer het percentage vaste huisartsen met patiënten op naam naar fte-rato in de loop van de tijd.*

Praktijkdoel 3: Patiëntendossiers in het HIS tonen een pop-up met de naam van de vaste huisarts

Met de pop-up worden assistenten, huisartsen en andere zorgverleners gewezen op de naam van de vaste huisarts. Zo kan bijvoorbeeld een afspraak worden ingepland bij deze huisarts, om de persoonlijke continuïteit te bevorderen.

Voorgestelde aanpak en activiteiten:

Voor deze aanpak is het noodzakelijk dat patiënten op naam staan in het HIS.

- ☐ Stel vast welke medewerkers u wilt betrekken bij het praktijkdoel. Registreer en communiceer dit naar de betrokken medewerkers.
- ☐ Bepaal hoeveel patiëntendossiers een pop-up moeten bevatten om aan het praktijkdoel te voldoen.
    - o *Bijvoorbeeld: alle patiëntendossiers of een selectie van de dossiers moeten een pop-up bevatten.*
- ☐ Leg vast wanneer u dit praktijkdoel bereikt wilt hebben.
- ☐ Bekijk, indien nodig, hoe pop-ups kunnen worden gerealiseerd in uw HIS.
    - o *In alle in Nederland verkrijgbare HIS'en kunt u pop-ups toevoegen aan de patiëntendossiers. Weet u niet hoe dit moet? Neem dan contact op met de helpdesk van uw HIS.*
- ☐ Bepaal aan welke patiëntendossiers u een pop-up wilt toevoegen.
    - o *Bijvoorbeeld: voeg een pop-toe aan de dossiers van alle nieuwe patiënten of alleen aan die van spreekuurbezoekers. Of identificeer patiëntendossiers via HIS-zoekopdrachten op bepaalde kenmerken, zoals patiëntnummer, postcode of achternaam.*
    - o *Hebt u weinig tijd of personeel om pop-ups toe te voegen? Overweeg dan bepaalde patiëntengroepen te prioriteren, zoals 65+-patiënten.*
- ☐ Overleg of bepaal wie, wanneer de pop-ups toevoegt aan de geselecteerde patiëntendossiers in het HIS.
- ☐ Wijs assistenten op het nut van de pop-up en instrueer hen om bij het maken van een afspraak altijd het patiëntendossier in het HIS te openen.
- ☐ Bedenk hoe u kunt waarborgen dat de betrokken medewerkers (blijven) handelen naar de pop-up.
- ☐ Is er sprake van 'pop-upmoeheid' bij de zorgverleners van uw praktijk? Bepaal dan welke pop-ups de meeste waarde hebben en verwijder de onnodige pop-ups.
- ☐ Besluit hoe en wanneer u de uitkomst van deze verbetersuggestie wilt monitoren.
    - o *Bijvoorbeeld: registreer het percentage patiëntendossiers dat een pop-up bevat met de naam van de vaste huisarts.*

Praktijkdoel 4: Assistenten plannen afspraken bij voorkeur in bij de vaste huisarts

Assistenten geven bij het inplannen van afspraken voorrang aan een afspraak bij de vaste huisarts. Hierdoor ziet de patiënt vaker zijn of haar vaste zorgverlener, wat de persoonlijke continuïteit vergroot.

Voorgestelde aanpak en activiteiten:

Voor deze aanpak is het noodzakelijk dat patiënten op naam staan in het HIS.

- ☐ Stel vast welke medewerkers u wilt betrekken bij het praktijkdoel. Registreer en communiceer dit naar de betrokken medewerkers.
- ☐ Bepaal in welke mate de assistenten patiënten bij hun vaste huisartsen moeten inplannen om het praktijkdoel te bereiken.
- ☐ Leg vast wanneer u dit praktijkdoel bereikt wilt hebben.
- ☐ Bespreek met uw collega huisartsen hoe wordt omgegaan met spoedeisende consulten.
- ☐ Instrueer de assistenten om bij het inplannen van afspraken altijd eerst in het HIS te kijken wie de vaste huisarts van de patiënt is. De assistent probeert vervolgens de afspraak bij deze huisarts in te plannen en niet bij een andere huisarts die eerder beschikbaar is, tenzij hier medische redenen voor zijn.
    - o *Is het bij het openen van het HIS niet direct duidelijk wie de vaste huisarts is? Dan kunt u overwegen om een pop-up met de naam van de vaste huisarts toe te voegen aan de patiëntendossiers in het HIS.*
- ☐ Bedenk hoe u kunt waarborgen dat de assistenten (blijven) handelen conform uw instructies.
    Bijvoorbeeld:
    - o *U kunt de instructies periodiek herhalen.*
    - o *Evalueer de manier van plannen van assistenten in een apart overleg.*
- ☐ Overweeg om patiënten te informeren over de soms langere wachttijd voor een afspraak bij de huisarts.
- ☐ Besluit hoe en wanneer u de uitkomst van deze verbetersuggestie wilt monitoren.
    Bijvoorbeeld:
    - o *Laat de assistenten of huisartsen een dagboek bijhouden gedurende 1 week per maand. In dit dagboek turven zij hoeveel afspraken ze hebben gemaakt en hoe vaak het gelukt is om deze in te plannen bij de vaste huisarts.*
    - o *De huisarts kijkt gedurende een werkweek aan het einde van de dag de spreekuuragenda na en telt het aantal consulten met eigen patiënten. Dit aantal wordt gedeeld door het totale aantal (eigen) consulten van die dag.*

Praktijkdoel 5: Assistenten plannen patiënten met complexe problemen bij voorkeur in bij hun vaste huisarts

Patiënten met complexe problemen hebben meer baat bij persoonlijke continuïteit. Wanneer assistenten bij het inplannen van afspraken voorrang geven aan een afspraak bij de vaste huisarts, ziet de patiënt vaker zijn of haar eigen zorgverlener. Hierdoor neemt de persoonlijke continuïteit toe.

Voorgestelde aanpak en activiteiten:

Voor deze aanpak is het noodzakelijk dat patiënten op naam staan in het HIS.

- ☐ Stel vast welke medewerkers u wilt betrekken bij het praktijkdoel. Registreer en communiceer dit naar de betrokken medewerkers.
- ☐ Bepaal in welke mate de assistenten patiënten met complexe problemen bij hun vaste huisarts moeten inplannen om het praktijkdoel te bereiken.
- ☐ Leg vast wanneer u dit praktijkdoel bereikt wilt hebben.
- ☐ Bepaal de definitie van een 'complex probleem'.
    - *Complexiteit wordt bepaald door diverse factoren. Denk bijvoorbeeld aan chronische psychische klachten, multimorbiditeit, een nadelige sociale situatie, zorgmijdend gedrag of een combinatie van deze factoren.*
    - *U kunt bepaalde ICPC-codes, of combinaties van meerdere ICPC-codes, gebruiken om patiënten met complexe problemen te identificeren.*
- ☐ Bedenk hoe de assistenten patiënten met complexe problemen kunnen identificeren.
- ☐ Bespreek met uw collega huisartsen hoe wordt omgegaan met spoedeisende consulten van complexe patiënten.
- ☐ Instrueer de assistenten om bij het inplannen van afspraken na te gaan of er sprake is van een complexe hulpvraag. De assistent kijkt dan in het HIS om na te gaan wie de vaste huisarts van de patiënt is. De assistent probeert vervolgens de afspraak bij deze huisarts in te plannen en niet bij een andere huisarts die eerder beschikbaar is, tenzij hier medische redenen voor zijn.
    - *Wanneer de patiënt een afspraak wil maken voor een niet-complex probleem, kan hij of zij daarvoor terecht bij iedere huisarts.*
    - *Is het bij het openen van het HIS niet direct duidelijk wie de vaste huisarts is? Dan kunt u overwegen om een pop-up met de naam van de vaste huisarts toe te voegen aan de patiëntendossiers in het HIS.*
- ☐ Bedenk hoe u kunt waarborgen dat de assistenten (blijven) handelen conform uw instructies.

    Bijvoorbeeld:
    - *U kunt bijvoorbeeld de instructies periodiek herhalen.*
    - *Evalueer de manier van plannen van assistenten in een apart overleg.*
- ☐ Overweeg om patiënten te informeren over de soms langere wachttijd voor een afspraak bij de huisarts.
- ☐ Besluit hoe en wanneer u de uitkomst van deze verbetersuggestie wilt monitoren.

    Bijvoorbeeld:
    - *Laat de assistenten of huisarts een dagboek bijhouden gedurende 1 week per maand. In dit dagboek turven zij hoeveel afspraken ze hebben gemaakt en hoe vaak het gelukt is om deze in te plannen bij de vaste huisarts.*
    - *De huisarts kijkt gedurende een werkweek aan het einde van de dag de spreekuuragenda na en telt het aantal consulten met eigen complexe patiënten. Dit aantal wordt gedeeld door het totale aantal (eigen) consulten met complexe patiënten van die dag. Dit getal wordt gemonitord.*

Praktijkdoel 6: Telefonische vervolgconsulten voor een ziekte-episode worden gedaan door de vaste huisarts

Door vervolgconsulten in te plannen bij de vaste huisarts, spreekt de patiënt zijn of haar vaste zorgverlener frequenter en kan de huisarts beter aanhaken bij het vorige consult. Hierdoor leren huisarts en patiënt elkaar beter kennen, wat bijdraagt aan de persoonlijke continuïteit.

Voorgestelde aanpak en activiteiten:

Voor deze aanpak is het noodzakelijk dat patiënten op naam staan in het HIS.

- ☐ Stel vast welke medewerkers u wilt betrekken bij het praktijkdoel. Registreer en communiceer dit naar de betrokken medewerkers.
- ☐ Bepaal in welke mate de assistenten telefonische vervolgconsulten voor één ziekte-episode bij de vaste huisarts moeten inplannen om het praktijkdoel te bereiken.
- ☐ Leg vast wanneer u dit praktijkdoel bereikt wilt hebben.
- ☐ Bespreek met de betrokken medewerkers hoe wordt omgegaan met spoedeisende telefonische vervolgconsulten.
- ☐ Instrueer de assistenten om bij het inplannen van telefonische vervolgconsulten altijd eerst in het HIS te kijken wie de vaste huisarts van de patiënt is. De assistent probeert vervolgens een telefonisch vervolgconsult bij deze huisarts in te plannen en niet bij een andere huisarts die eerder beschikbaar is, tenzij hier medische redenen voor zijn.
    - *Is het bij het openen van het HIS niet direct duidelijk wie de vaste huisarts is? Dan kunt u overwegen om een pop-up met de naam van de vaste huisarts toe te voegen aan de patiëntendossiers in het HIS.*
- ☐ Bedenk hoe u kunt waarborgen dat de assistenten (blijven) handelen conform uw instructies.
  Bijvoorbeeld:
    - *U kunt de instructies periodiek herhalen.*
    - *Evalueer de manier van plannen van assistenten in een apart overleg.*
- ☐ Overweeg om patiënten te informeren over de soms langere wachttijd voor een afspraak bij de huisarts.
- ☐ Besluit hoe en wanneer u de uitkomst van deze verbetersuggestie wilt monitoren.
  Bijvoorbeeld:
    - *Laat de assistenten of huisarts een dagboek bijhouden gedurende 1 week per maand. In dit dagboek turven zij hoeveel telefonisch vervolgconsulten ze hebben gemaakt en hoe vaak het gelukt is om deze in te plannen bij de vaste huisarts.*
    - *De huisarts kijkt gedurende een werkweek aan het einde van dag de spreekuuragenda na en telt het aantal telefonische vervolgconsulten met eigen patiënten. Dit aantal wordt gedeeld door het totale aantal (eigen) telefonische vervolgconsulten van die dag. Dit getal wordt gemonitord.*

Praktijkdoel 7: Eén probleem, één huisarts

Om patiënten zo veel mogelijk terug te laten komen bij dezelfde huisarts, worden consulten voor één ziekte-episode bij voorkeur bij dezelfde huisarts ingepland. Hierdoor bouwt de patiënt een relatie op met deze huisarts, wat de persoonlijke continuïteit vergroot.

Voorgestelde aanpak en activiteiten:

- ☐ Stel vast welke medewerkers u wilt betrekken bij het praktijkdoel. Registreer en communiceer dit naar de betrokken medewerkers.
- ☐ Bepaal in welke mate de assistenten één probleem bij één huisarts moeten inplannen om het praktijkdoel te bereiken.
- ☐ Leg vast wanneer u dit praktijkdoel bereikt wilt hebben.
- ☐ Bespreek met uw collega huisartsen wanneer een patiënt beter zijn of haar vaste huisarts ziet in plaats van de eerst geconsulteerde huisarts.
- ☐ Bespreek met alle betrokken medewerkers hoe wordt omgegaan met spoedeisende vervolgconsulten.
- ☐ Instrueer de assistenten om bij het inplannen van een afspraak te vragen of de patiënt al eerder bij een huisarts is geweest voor het actuele probleem en zo ja, welke huisarts dit is. Als de patiënt dit niet weet, dient de assistent in het HIS te kijken om te zien of de patiënt voor dit probleem recent bij een huisarts is geweest.
  - *Is het bij het openen van het HIS niet direct duidelijk wie de vaste huisarts is? Dan kunt u overwegen om een pop-up met de naam van de vaste huisarts toe te voegen aan de patiëntendossiers in het HIS.*
- ☐ Bedenk hoe u kunt waarborgen dat de assistenten (blijven) handelen conform uw instructies.
  Bijvoorbeeld:
  - *U kunt de instructies periodiek herhalen.*
  - *Evalueer de manier van plannen van assistenten in een apart overleg.*
  - *Patiënten moeten soms langer wachten op een afspraak. Dit vraagt ook om draagvlak bij en aanpassing van de assistenten.*
- ☐ Besluit hoe en wanneer u de uitkomst van deze verbetersuggestie wilt monitoren.
  Bijvoorbeeld:
  - *Laat de assistenten of huisarts een dagboek bijhouden gedurende 1 week per maand. In dit dagboek turven zij hoeveel afspraken ze hebben gemaakt en hoe vaak het gelukt is om deze in te plannen bij de huisarts die de patiënt als eerst heeft gezien.*
  - *De huisarts kijkt gedurende een werkweek aan het einde van dag de spreekuuragenda na en telt het aantal vervolgconsulten met patiënten die hij of zij ook als eerste heeft gezien. Dit aantal wordt gedeeld door het totale aantal (eigen) vervolgconsulten van die dag. Dit getal wordt gemonitord.*

Praktijkdoel 8: Huisbezoeken bij huisgebonden patiënten worden gedaan door één (of twee) vaste huisarts(en)

Bij het openen van het dossier van een patiënt zorgt u ervoor dat het HIS een pop-up toont bij patiënten die tijdelijk of langdurig niet in staat zijn om naar de praktijk te komen. Deze pop-up bevat afspraken over welke vaste huisarts(en) huisbezoeken aflegt/afleggen bij deze patiënt. Hierdoor ziet de patiënt maar één of twee vaste huisartsen, wat de persoonlijke continuïteit vergroot.

Voorgestelde aanpak en activiteiten:

- ☐ Stel vast welke medewerkers u wilt betrekken bij het praktijkdoel. Registreer en communiceer dit naar de betrokken medewerkers.
- ☐ Bepaal in welke mate huisbezoeken bij huisgebonden patiënten door één of twee vaste huisartsen moeten worden gedaan om het praktijkdoel te bereiken.
- ☐ Leg vast wanneer u dit praktijkdoel bereikt wilt hebben.
- ☐ Overleg met uw huisartsenteam welke patiënten onder de noemer 'huisgebonden' vallen en hoe zij kunnen worden geïdentificeerd in het HIS.
- ☐ Bepaal welke huisartsen visites zullen afleggen bij huisgebonden patiënten. Denk bijvoorbeeld aan de vaste huisarts van een patiënt en diens eventuele duopartner.
- ☐ Bepaal hoe in het HIS duidelijk wordt gemaakt welke vaste huisarts(en) bij deze patiënten visites mag/mogen afleggen.
    - *Bijvoorbeeld: zet huisgebonden patiënten op naam of voeg een pop-up toe aan hun dossier in het HIS met de naam/namen van de huisarts(en) die visites mag/mogen afleggen.*
- ☐ Bepaal wie, wanneer de patiënten identificeert en hoe dit moet worden geregistreerd in het HIS.
- ☐ Instrueer de assistenten om bij het inplannen van afspraken van visites het HIS te openen en te handelen conform de HIS-instructie bij huisgebonden patiënten.
- ☐ Bedenk hoe u kunt waarborgen dat de assistenten (blijven) handelen conform uw instructies.
  Bijvoorbeeld:
    - *U kunt bijvoorbeeld de instructies periodiek herhalen.*
    - *Evalueer de manier van plannen van assistenten in een apart overleg.*
    - *Patiënten moeten soms langer wachten op een afspraak. Dit vraagt ook om draagvlak bij en aanpassing van de assistenten.*
- ☐ Besluit hoe en wanneer u de uitkomst van deze verbetersuggestie wilt monitoren.
  Bijvoorbeeld:
    - *Laat de assistenten of huisarts een dagboek bijhouden gedurende 1 week per maand. In dit dagboek turven zij bij hoeveel huisgebonden patiënten zij een visite hebben ingepland bij de vaste huisarts.*
    - *De huisarts kijkt gedurende een werkweek aan het einde van dag de spreekuuragenda na en telt het aantal huisbezoeken bij eigen huisgebonden patiënten. Dit aantal wordt gedeeld door het totale aantal huisbezoeken bij huisgebonden patiënten van die dag. Dit getal wordt gemonitord.*

Praktijkdoel 2: Herhaalrecepten worden bij voorkeur uitgeschreven door de vaste huisarts

Het voorschrijven van herhaalrecepten door een huisarts die de patiënt kent kan leiden tot minder medicatiefouten en minder onterechte herhaalrecepten (bijvoorbeeld door te lang voorschrijven van benzodiazepines of opiaten).

Voorgestelde aanpak en activiteiten:

- ☐ Stel vast welke medewerkers u wilt betrekken bij het praktijkdoel. Registreer en communiceer dit naar de betrokken medewerkers.
- ☐ Bepaal in welke mate herhaalrecepten door de vaste huisarts moeten worden uitgeschreven om het praktijkdoel te bereiken.
    - *Bijvoorbeeld: maak een keuze om alleen herhaalrecepten van één specifieke groep van geneesmiddelen (zoals benzodiazepines of opiaten) door de vaste huisarts uit te laten schrijven.*
- ☐ Leg vast wanneer u dit praktijkdoel bereikt wilt hebben.
- ☐ Overleg met uw huisartsenteam hoe u kunt faciliteren dat herhaalrecepten standaard door de vaste huisarts worden voorgeschreven.
- ☐ Leg afspraken vast over hoe spoedeisende herhaalrecepten worden afgehandeld als de vaste huisarts niet aanwezig is.
- ☐ Stel een termijn vast waarbinnen niet-spoedeisende herhaalrecepten verwerkt moeten zijn.
- ☐ Stel het hele huisartsenteam op de hoogte van de nieuwe werkwijze omtrent het uitschrijven van herhaalrecepten.
- ☐ Overweeg om patiënten te informeren over de soms langere wachttijd voor een niet-spoedeisend herhaalrecept en hun te verzoeken tijdig om een herhaalrecept te vragen.
- ☐ Besluit hoe en wanneer u de uitkomst van deze verbetersuggestie wilt monitoren.
    - *Bijvoorbeeld: registreer het percentage herhaalrecepten uitgeschreven door de eigen huisarts ten opzichte van het totale aantal herhaalrecepten van een patiënt gedurende een tijdsperiode.*

Praktijkdoel 10: Laboratoriumuitslagen worden beoordeeld en teruggekoppeld door de aanvragend huisarts

De aanvragend huisarts weet waarom hij of zij bepaalde diagnostiek heeft aangevraagd en kan de uitslagen ervan daarom beter interpreteren in de context van het actuele probleem van de patiënt. Door de laboratoriumuitslagen ook zelf terug te koppelen naar de patiënt, is het voor de aanvragend huisarts gemakkelijker om persoonsgerichte zorg te leveren en het persoonlijke contact te bevorderen.

<u>Voorgestelde aanpak en activiteiten</u>:

- ☐ Stel vast welke medewerkers u wilt betrekken bij het praktijkdoel. Registreer en communiceer dit naar de betrokken medewerkers.
- ☐ Bepaal in welke mate laboratoriumuitslagen moeten worden afgehandeld door de aanvragend huisarts om het praktijkdoel te bereiken.
- ☐ Leg vast wanneer u dit praktijkdoel bereikt wilt hebben.
- ☐ Stem met uw huisartsenteam af hoe u de werkwijze van de praktijk zo kunt aanpassen dat de huisarts die een laboratoriumonderzoek aanvraagt, ook de terugkoppeling naar de patiënt doet en, indien nodig, zelf een vervolgafspraak inplant.
- ☐ Maak afspraken over de situaties waarin een uitzondering moet worden gemaakt. Bijvoorbeeld: bij spoeddiagnostiek of afwijkende uitslagen.
- ☐ Stel een termijn vast waarbinnen niet-spoedeisende laboratoriumuitslagen verwerkt moeten zijn.
    - *Zijn er in uw praktijk huisartsen die minder dan 3 dagen per week werken, of werkt u met veel wisselende waarnemers? Besteed hier dan extra aandacht aan bij het maken van afspraken over de beoordeling van laboratoriumuitslagen.*
- ☐ Overweeg om patiënten te informeren over de soms langere wachttijd voor niet-spoedeisende uitslagen.
- ☐ Bedenk hoe u kunt waarborgen dat de assistenten (blijven) handelen conform uw instructies.
  Bijvoorbeeld:
    - *U kunt de instructies periodiek herhalen.*
    - *U kunt een overleg inplannen met de assistenten waarin u de manier van omgang met laboratoriumuitslagen evalueert*.
- ☐ Besluit hoe en wanneer u de uitkomst van deze verbetersuggestie wilt monitoren.
    - *Bijvoorbeeld: registreer gedurende een week aan het einde van de dag hoeveel laboratoriumuitslagen in het HIS zijn afgehandeld door de aanvragend huisarts en deel dit door het totale aantal afgehandelde laboratoriumuitslagen van die dag.*

Praktijkdoel 11: Parttime-huisartsen werken samen als duodokters

Patiënten van huisartsen die parttime werken, krijgen een tweede vaste huisarts. Patiënten zien hierdoor minder verschillende hulpverleners en kunnen zo een betere band opbouwen met hun huisartsen.

Voorgestelde aanpak en activiteiten:

- ☐ Stel vast welke medewerkers u wilt betrekken bij het praktijkdoel. Registreer en communiceer dit naar de betrokken medewerkers.
- ☐ Leg vast wanneer u dit praktijkdoel bereikt wilt hebben.
- ☐ Maak een plan waarin u bepaalt welke huisartsen die parttime werken (< 0,8 fte) met wie van de andere full- of parttime werkende huisartsen een duo kunnen vormen.
    - *Let op dat deze duodokters gezamenlijk een beschikbaarheid van minstens 4 dagen voor patiëntenzorg kunnen garanderen.*
- ☐ Bespreek uw voorstel met uw collega-huisartsen en leg vast welke huisartsen aan elkaar gekoppeld worden.
- ☐ Bepaal wie, wanneer en hoe de duo's vastlegt in het HIS.
    - *Hebt u weinig tijd of personeel om alle patiënten van een duo tijdig op naam te zetten? Voer de wijzigingen dan stapsgewijs in of prioriteer bepaalde patiëntengroepen, zoals 65+-patiënten.*
- ☐ Instrueer de assistenten om bij het maken van afspraken in het HIS te kijken wie eventueel de duodokters van de patiënt zijn. De assistenten maken bij voorkeur een afspraak bij een van de twee vaste duodokters.
    - *U kunt overwegen om in het HIS een pop-up toe te voegen met de namen van de vaste duodokters.*
- ☐ Besluit, eventueel in overleg, wanneer en in welke mate patiënten inspraak krijgen bij de aanstelling van een tweede vast aanspreekpunt.
    - *Houd er rekening mee dat sommige patiënten het liefst maar één huisarts willen bezoeken en geen andere.*
- ☐ Communiceer naar de patiënten dat zij een tweede vast aanspreekpunt hebben en hoe zij inspraak kunnen uitoefenen op de artsenkeuze.
- ☐ Bedenk hoe u kunt waarborgen dat de assistenten (blijven) handelen conform uw instructies.
  Bijvoorbeeld:
    - *U kunt de instructies periodiek herhalen.*
    - *Evalueer de manier van plannen van assistenten in een apart overleg.*
- ☐ Besluit hoe en wanneer u de uitkomst van deze verbetersuggestie wilt monitoren.
    - *Bijvoorbeeld: maak een lijst met de namen van alle vaste huisartsen, hun fte en of ze een duo gevormd hebben.*

Praktijkdoel 12: Patiënten met complexe problemen hebben twee vaste huisartsen

Patiënten met complexe problemen hebben meer baat bij persoonlijke continuïteit. Door te werken met maximaal twee vaste huisartsen, zien patiënten minder verschillende hulpverleners en kunnen zij een betere band opbouwen met hun huisartsen.

<u>Voorgestelde aanpak en activiteiten</u>:

- ☐ Stel vast welke medewerkers u wilt betrekken bij het praktijkdoel. Registreer en communiceer dit naar de betrokken medewerkers.
- ☐ Leg vast wanneer u dit praktijkdoel bereikt wilt hebben.
- ☐ Bepaal de definitie van een ‘complex probleem’.
    - o *Complexiteit wordt bepaald door diverse factoren. Denk bijvoorbeeld aan chronische psychische klachten, multimorbiditeit, een nadelige sociale situatie, zorgmijdend gedrag of een combinatie van deze factoren.*
    - o *U kunt bepaalde ICPC-codes, of combinaties van meerdere ICPC-codes, gebruiken om patiënten met complexe problemen te identificeren.*
- ☐ Bepaal wie, wanneer de patiënten met complexe problemen identificeert in het HIS.
- ☐ Wijs in overleg met uw collega-huisartsen voor iedere patiënt met een complex probleem een extra huisarts aan, naast de vaste huisarts. Deze tweede huisarts is het tweede vaste aanspreekpunt voor deze patiënt.
    - o *Let op dat deze twee huisartsen gezamenlijk een beschikbaarheid van minstens 4 dagen voor patiëntenzorg kunnen garanderen.*
- ☐ Bepaal wie, wanneer en hoe in het HIS registreert dat deze patiënten een tweede vast aanspreekpunt hebben.
- ☐ Instrueer de assistenten om bij het maken van afspraken in het HIS te kijken wie de vaste huisartsen van de patiënt zijn. De assistenten maken bij voorkeur een afspraak bij een van de twee vaste huisartsen.
    - o *U kunt overwegen om in het HIS een pop-up toe te voegen met de namen van de vaste huisartsen.*
- ☐ Besluit, eventueel in overleg, wanneer en in welke mate patiënten inspraak krijgen bij de aanstelling van een tweede vast aanspreekpunt.
    - o *Houd er rekening mee dat sommige patiënten het liefst maar één huisarts willen bezoeken en geen andere.*
- ☐ Communiceer naar de patiënten dat zij een tweede vast aanspreekpunt hebben en hoe zij inspraak kunnen uitoefenen op de artsenkeuze.
- ☐ Bedenk hoe u kunt waarborgen dat de assistenten (blijven) handelen conform uw instructies.
  Bijvoorbeeld:
    - o *U kunt bijvoorbeeld de instructies periodiek herhalen.*
    - o *Evalueer de manier van plannen van assistenten in een apart overleg.*
- ☐ Besluit hoe en wanneer u de uitkomst van deze verbetersuggestie wilt monitoren.
  Bijvoorbeeld:
    - o *Maak een lijst van patiënten met complexe problemen en geef per patiënt aan welke twee huisartsen de vaste aanspreekpunten zijn.*
    - o *Registreer via HIS-zoekopdrachten hoeveel patiënten met complexe problemen een tweede vaste huisarts toegewezen hebben gekregen.*

Praktijkdoel 13: Duodokters hebben periodieke overlegmomenten

Parttime werkende huisartsen die patiënten delen, hebben periodiek overleg. Hierdoor wordt de behandeling van patiënten afgestemd en worden individuele observaties en overwegingen overlegd. Dit leidt tot meer continue zorg.

<u>Voorgestelde aanpak en activiteiten</u>:

Deze aanpak gaat ervan uit dat u werkt met duodokters.

- ☐ Stel vast welke medewerkers u wilt betrekken bij het praktijkdoel. Registreer en communiceer dit naar de betrokken medewerkers.
- ☐ Bepaal de frequentie en duur van de periodieke overlegmomenten.
  - o *Afhankelijk van de werkdagen van de duodokters kan het moeilijk zijn een gezamenlijk overlegmoment te vinden.*
- ☐ Leg vast wanneer u dit praktijkdoel bereikt wilt hebben.
- ☐ Bepaal wat de inhoud van de overlegmomenten wordt.
  - o *Houd er rekening mee dat extra overlegmomenten ten koste kunnen gaan van tijd voor andere werkzaamheden, zoals spreekuurtijd.*
- ☐ Bepaal of de duodokters tijdens hun overlegmoment gestoord mogen worden voor telefonisch overleg en wat het beleid is bij spoedgevallen tijdens het overleg.
- ☐ Besluit hoe en wanneer u de uitkomst van deze verbetersuggestie wilt monitoren.
  Bijvoorbeeld:
  - o *Aantal overlegmomenten tussen duopartners per maand.*
  - o *Aantal ondernomen acties aan de hand van de overlegmomenten.*

Groot L, *et al. BMJ Open* 2024; 14:e078169. doi: 10.1136/bmjopen-2023-078169

Praktijkdoel 14: Iedere huisarts biedt nieuw ingeschreven 65+-patiënten een kennismakingsgesprek aan

Patiënten waarderen een proactieve houding van hun huisarts. Een kennismakingsgesprek kan het opbouwen van de vertrouwensband tussen huisarts en patiënt vergemakkelijken.

Voorgestelde aanpak en activiteiten:

- ☐ Stel vast welke medewerkers u wilt betrekken bij het praktijkdoel. Registreer en communiceer dit naar de betrokken medewerkers.
- ☐ Leg vast wanneer u dit praktijkdoel bereikt wilt hebben.
- ☐ Bepaal welke 65+-patiënten uw praktijk gaat uitnodigen voor een kennismakingsgesprek.
    - *Bijvoorbeeld: nodig alle nieuwe 65+-patiënten uit, alle nieuwe 65+-patiënten van de afgelopen 3 maanden of alleen 65+-patiënten met chronische ziekten.*
    - *Als uw praktijk veel nieuwe 65+-patiënten heeft, kan de tijd voor reguliere consulten afnemen en de wachttijd oplopen.*
- ☐ Bepaal wie, wanneer en hoe de patiënten uitnodigt voor de kennismakingsgesprekken.
    - *Overweeg patiënten bij de uitnodiging te wijzen op de Thuisarts-informatie Kennismakingsgesprek met de huisarts.*
- ☐ Besluit hoe en wanneer u de uitkomst van deze verbetersuggestie wilt monitoren.
    - *Bijvoorbeeld: registreer het percentage nieuwe 65+-patiënten dat een kennismakingsgesprek is aangeboden ten opzichte van het totale aantal 65+-patiënten gedurende een jaar.*

Praktijkdoel 15: Iedere huisarts heeft periodiek telefonisch contact met patiënten met complexe problemen

Patiënten waarderen een proactieve houding van hun huisarts. Door periodiek contact op te nemen, houdt de huisarts de vinger aan de pols bij patiënten met complexe problemen. Het grotere aantal contacten en de proactieve houding komen de persoonlijke continuïteit ten goede.

Voorgestelde aanpak en activiteiten:

- ☐ Stel vast welke medewerkers u wilt betrekken bij het praktijkdoel. Registreer en communiceer dit naar de betrokken medewerkers.
- ☐ Leg vast wanneer u dit praktijkdoel bereikt wilt hebben.
- ☐ Bepaal de definitie van een 'complex probleem'.
    - *Complexiteit wordt bepaald door diverse factoren. Denk bijvoorbeeld aan chronische psychische klachten, multimorbiditeit, een nadelige sociale situatie, zorgmijdend gedrag of een combinatie van deze factoren.*
    - *U kunt bepaalde ICPC-codes, of combinaties van meerdere ICPC-codes, gebruiken om patiënten met complexe problemen te identificeren.*
    - *Als veel patiënten als 'complex' zijn aangemerkt, kan dit praktijkdoel te tijdsintensief worden. Om het doel dan haalbaarder te maken, kunt u overwegen een smallere definitie van 'complex probleem' te hanteren.*
- ☐ Bepaal wie, wanneer en op welke manier de patiënten met complexe problemen identificeert in het HIS.
- ☐ Stel een lijst op van patiënten met complexe problemen en hun vaste huisarts.
- ☐ Overleg met uw collega-huisartsen hoe de telefonische contacten met deze patiënten worden georganiseerd. Bepaal hoe vaak, wanneer en hoelang getelefoneerd moet worden. Bespreek ook wat de gespreksonderwerpen van deze contacten zijn.
- ☐ Besluit hoe en wanneer u de uitkomst van deze verbetersuggestie wilt monitoren.
    Bijvoorbeeld:
    - *Registreer het aantal huisartsen in de praktijk met een bellijst van complexe patiënten.*
    - *Registreer het aantal telefonische contacten op initiatief van de huisarts bij complexe patiënten per tijdsinterval.*

Groot L, et al. BMJ Open 2024; 14:e078169. doi: 10.1136/bmjopen-2023-078169

Praktijkdoel 16: De vaste huisarts neemt standaard contact op met de patiënt bij thuiskomst na een ziekenhuisopname voor een ingrijpende behandeling of gebeurtenis voor de patiënt

De meeste patiënten hebben behoefte aan een contactmoment met hun eigen huisarts bij een ingrijpende gebeurtenis. Zij hebben het gevoel dat de eigen huisarts hun algehele medische conditie en persoonlijke situatie goed kent. Een contactmoment na een ziekenhuisopname voor een ingrijpende behandeling of gebeurtenis laat interesse van de huisarts zien en versterkt de vertrouwensband tussen huisarts en patiënt.

<u>Voorgestelde aanpak en activiteiten</u>:

- ☐ Stel vast welke medewerkers u wilt betrekken bij het praktijkdoel. Registreer en communiceer dit naar de betrokken medewerkers.
- ☐ Leg vast wanneer u dit praktijkdoel bereikt wilt hebben.
- ☐ Leg vast wanneer u dit praktijkdoel bereikt wilt hebben.
- ☐ Bepaal in overleg met uw huisartsenteam de definitie van een ‘ingrijpende gebeurtenis’.
    - *De definitie van een ‘ingrijpende gebeurtenis’ kan per patiënt verschillen. U kunt overwegen om een lijst samen te stellen van gebeurtenissen die altijd ingrijpend zijn, zoals de diagnose van een ernstige ziekte.*
- ☐ Overleg met uw huisartsenteam hoe contact wordt opgenomen met de patiënt na kennisname van ontslag uit het ziekenhuis voor een ingrijpende gebeurtenis.
- ☐ Leg vast binnen welke termijn er contact moet zijn geweest.
    - *Wanneer er problemen zijn met de communicatie vanuit het ziekenhuis, kan het langer duren voordat contact kan worden opgenomen na ontslag.*
- ☐ Instrueer de medewerker(s) die de post verwerkt/verwerken dat ontslagberichten van patiënten die waren opgenomen voor een ingrijpende gebeurtenis bekend moeten worden gemaakt bij de vaste huisarts van die patiënt.
- ☐ Besluit hoe en wanneer u de uitkomst van deze verbetersuggestie wilt monitoren.
  Bijvoorbeeld:
    - *Noteer de afspraken over het opnemen van contact met een patiënt na ziekenhuisopname voor een ingrijpende gebeurtenis.*
    - *Laat huisartsen gedurende een tijdsperiode turven hoe vaak zij op eigen initiatief een contactmoment hebben ingepland met een patiënt na ziekenhuisopname voor een ingrijpende gebeurtenis.*
    - *Registreer via HIS-zoekopdrachten het percentage patiënten dat een contactmoment heeft gehad na ziekenhuisopname voor een ingrijpende gebeurtenis.*

Praktijkdoel 17: Identificatie van patiënten met een lage continuïteit

Periodiek wordt een zoekopdracht uitgevoerd om patiënten met een lage continuïteit te identificeren. De huisarts kan zo monitoren welke patiënten een lagere persoonlijke continuïteit hebben en daar continuïteitsbevorderende acties aan verbinden.

Voorgestelde aanpak en activiteiten:

- ☐ Stel vast welke medewerkers u wilt betrekken bij het praktijkdoel. Registreer en communiceer dit naar de betrokken medewerkers.
- ☐ Bepaal wie, wanneer de patiëntengegevens extraheert uit het HIS en de UPC-index berekent.
- ☐ Bepaal voor welke patiëntenpopulatie u de UPC-index wilt berekenen. Bijvoorbeeld: 65+-patiënten of patiënten met multimorbiditeit.
- ☐ Zoek de patiëntenpopulatie op in het HIS. Extraheer de consultatiegegevens van deze patiënten. Bepaal hierbij hoeveel consulten zij hebben gehad gedurende 2 jaar, bij welke huisartsen deze consulten waren en wie de vaste huisarts is.
    - *Alle HIS'en in Nederland, op TetraHIs na, ondersteunen de functionaliteit van zoekopdrachten. Weet u niet hoe u deze zoekopdracht moet uitvoeren? Neem dan contact op met de helpdesk van uw HIS.*
- ☐ Bereken de UPC-index door de gegevens van de patiënten en consulten in te voeren in de calculator.
    - *De UPC-index wordt berekend door het totale aantal bezoeken van een patiënt met de eigen (vaste) huisarts te delen door het totale aantal bezoeken van die patiënt aan een huisarts gedurende een periode. De schaal van de UPC-index loopt van 0-1, waarbij '0' staat voor minimale continuïteit en '1' voor perfecte continuïteit.*
    - *Ervaart u problemen met deze calculator? Neem dan contact op met Lex Groot.*
- ☐ Stel een lijst samen van patiënten met de laagste UPC-index.
    - *U kunt bijvoorbeeld alle patiënten met een UPC-index < 0,5 opnemen in de lijst of patiënten met een UPC-index behorende tot de laagste 10%.*
- ☐ Overleg met uw collega-huisartsen de lijst en bespreek welke acties hieraan worden verbonden en wanneer deze worden uitgevoerd.
  Bijvoorbeeld:
    - *Laat een assistent alle patiënten met een lage continuïteit uitnodigen voor een spreekuurbezoek.*
    - *Alle patiënten met een lage continuïteit worden gebeld door hun vaste huisarts.*
    - *Zet de patiënten met een lage continuïteit op naam van een huisarts.*
- ☐ Bepaal wanneer deze zoekopdracht en acties worden herhaald en wie dit gaat doen.
- ☐ Besluit hoe en wanneer u de uitkomst van deze verbetersuggestie wilt monitoren.
  Bijvoorbeeld:
    - *Is er een lijst van patiënten met een lage UPC-index en de naam van de huisarts die contact met hen gaat opnemen? Ja/nee*
    - *Monitor de UPC-index van alle patiënten in de loop van een jaar.*

Groot L, et al. BMJ Open 2024; 14:e078169. doi: 10.1136/bmjopen-2023-078169

Praktijkdoel 18: Patiënten worden via de praktijkwebsite structureel geïnformeerd over de personeelsbezetting en de werktijden en werkdagen van zorgverleners

Patiënten kunnen op de praktijkwebsite actuele informatie zien over welke zorgverleners op welke dagen in de praktijk werken. Zij kunnen zo zelf afwegen of hun klacht kan wachten op hun vaste huisarts, waardoor zij minder snel naar een waarnemer zullen gaan.

Voorgestelde aanpak en activiteiten:

- ☐ Stel vast welke medewerkers u wilt betrekken bij het praktijkdoel. Registreer en communiceer dit naar de betrokken medewerkers.
- ☐ Bepaal wie verantwoordelijk is voor het updaten van de praktijkwebsite en wanneer en hoe vaak hij of zij dit moet doen. Leg hierbij tevens vast welke actuele informatie over de zorgverleners op de website moet staan.
  - *Bijvoorbeeld: de website bevat informatie over actuele werkdagen, bereikbaarheid voor spreekuren, data van geplande afwezigheid, en vertrek of komst van personeel.*
  - *Is er tussendoor een personeelswijziging, bijvoorbeeld door vertrek, ziekte of zwangerschap van een medewerker? Pas dan de website zo spoedig mogelijk aan.*
- ☐ Bedenk hoe u ervoor kunt zorgen dat de praktijkwebsite elke 3 maanden geactualiseerd wordt.
- ☐ Besluit hoe en wanneer u de uitkomst van deze verbetersuggestie wilt monitoren.
  - *Bijvoorbeeld: ga periodiek na of de informatie op de praktijkwebsite actueel is.*

Praktijkdoel 19: Patiënten worden via een nieuwsbrief structureel geïnformeerd over wijzigingen in personeel en in werktijden en -dagen van zorgverleners

De praktijk stuurt een periodieke nieuwsbrief – digitaal of per post. Zo worden patiënten geïnformeerd over eventuele wijzigingen in de werktijden of -dagen van de zorgverleners en over eventuele personeelswijzigingen. Zij kunnen zo zelf afwegen of hun klacht kan wachten op hun vaste huisarts, waardoor zij minder snel naar een waarnemer zullen gaan.

Voorgestelde aanpak en activiteiten:

- ☐ Stel vast welke medewerkers u wilt betrekken bij het praktijkdoel. Registreer en communiceer dit naar de betrokken medewerkers.
- ☐ Bepaal wie, wanneer en hoe vaak de nieuwsbrief verstuurt. Bedenk ook op welke wijze de nieuwsbrief verstuurd gaat worden: per e-mail of post.
- ☐ Overleg welke informatie in de nieuwsbrief komt te staan en leg dit vast.
    - *Bijvoorbeeld: de nieuwsbrief bevat informatie over actuele werkdagen, bereikbaarheid voor spreekuren, data van geplande afwezigheid berichtgeving, en vertrek en komst van personeel.*
    - *Is er tussendoor een personeelswijziging, bijvoorbeeld door vertrek, ziekte of zwangerschap van een medewerker? Verstuur dan een extra nieuwsbrief.*
- ☐ Bied patiënten de mogelijkheid zich in of uit te schrijven voor deze nieuwsbrief (opt-in- of opt-outprocedure).
- ☐ Besluit hoe en wanneer u de uitkomst van deze verbetersuggestie wilt monitoren.
  Bijvoorbeeld:
    - *Is er een nieuwsbriefsjabloon voor actuele praktijkinformatie? Ja/nee*
      Registreer het aantal keer dat een nieuwsbrief verzonden is in een bepaalde periode

Praktijkdoel 20: Patiënten kunnen elektronische consulten aanvragen

De praktijk gaat e-consulten aanbieden aan patiënten. Hierdoor neemt de bereikbaarheid van de vaste huisarts voor de patiënten toe, en andersom. Patiënten kunnen zo gemakkelijker hun hulpvraag stellen aan een huisarts die hen kent.

Voorgestelde aanpak en activiteiten:

- ☐ Stel vast welke medewerkers u wilt betrekken bij het praktijkdoel. Registreer en communiceer dit naar de betrokken medewerkers.
- ☐ Leg vast wanneer u dit praktijkdoel bereikt wilt hebben.
- ☐ Neem contact op met een softwareleverancier om te vragen naar de mogelijkheden om e-consultatie te implementeren.
    - *Alle HIS-leveranciers ondersteunen een of meerdere patiëntenportalen met de mogelijkheid tot e-consultatie en integratie daarvan met het HIS en/of de praktijkwebsite. U kunt contact opnemen met uw HIS-leverancier voor meer informatie.*
    - *Software voor e-consultatie dient te voldoen aan de norm NEN 7510 en aan de Algemene verordening gegevensbescherming (AVG). Vraag hier expliciet naar bij het kiezen van een softwareleverancier voor e-consultatie.*
    - *Afhankelijk van de reeds bestaande ICT-infrastructuur en softwarepakketten kunnen er bijkomende kosten zijn voor het organiseren van e-consultatie.*
- ☐ Overleg met collega-huisartsen en assistenten over de organisatie, toepassing en inrichting van de e-consulten.
- ☐ Bepaal wie, wanneer en hoe de patiënten informeert over de mogelijkheid om een e-consult aan te vragen en hoe dit in zijn werk gaat.
- ☐ Leg vast wanneer u dit praktijkdoel bereikt wilt hebben.
- ☐ Besluit hoe en wanneer u de uitkomst van deze verbetersuggestie wilt monitoren.
    - *Bijvoorbeeld: kunnen patiënten e-consulten aanvragen? Ja/nee*

Praktijkdoel 21: Patiënten kunnen via internet afspraken maken bij een zorgverlener naar keuze

Patiënten kunnen via internet een afspraak maken bij hun vaste zorgverlener. Hierdoor is de bereikbaarheid van de praktijk groter dan wanneer ze dit telefonisch moeten doen. Ook kunnen patiënten eenvoudiger een afspraak maken bij een zorgverlener die zij kennen.

Voorgestelde aanpak en activiteiten:

- ☐ Stel vast welke medewerkers u wilt betrekken bij het praktijkdoel. Registreer en communiceer dit naar de betrokken medewerkers.
- ☐ Leg vast wanneer u dit praktijkdoel bereikt wilt hebben.
- ☐ Neem contact op met een softwareleverancier om te vragen naar de mogelijkheden om het maken van afspraken via internet te implementeren.
    - *Alle HIS-leveranciers ondersteunen een of meerdere patiëntenportalen met de mogelijkheid tot het maken van afspraken online en integratie daarvan met het HIS en/of de praktijkwebsite. U kunt contact opnemen met uw HIS-leverancier voor meer informatie.*
    - *Afhankelijk van de reeds bestaande ICT-infrastructuur en softwarepakketten kunnen er bijkomende kosten zijn voor het organiseren van het maken van afspraken online.*
- ☐ Bepaal wie, wanneer en hoe de patiënten informeert over de mogelijkheid om een afspraak online te maken en hoe dit in zijn werk gaat.
- ☐ Leg vast wanneer u dit praktijkdoel bereikt wilt hebben.
- ☐ Besluit hoe en wanneer u de uitkomst van deze verbetersuggestie wilt monitoren.

    Bijvoorbeeld:
    - *Kunnen patiënten online afspraken maken? Ja/nee*
    - *Registeren van het percentage consulten dat online is gemaakt per tijdseenheid*

Praktijkdoel 22: Huisartsen houden een telefonisch spreekuur voor eigen patiënten op werkdagen zonder vast spreekuur

Er is een telefonisch spreekuur voor eigen patiënten op werkdagen dat een huisarts geen vast spreekuur heeft. Dit bevordert de bereikbaarheid van de huisarts voor zijn of haar vaste patiënten. Het is hierdoor gemakkelijker voor patiënten om persoonlijk contact te hebben met hun eigen huisarts.

Voorgestelde aanpak en activiteiten:

- ☐ Stel vast welke medewerkers u wilt betrekken bij het praktijkdoel. Registreer en communiceer dit naar de betrokken medewerkers.
- ☐ Bepaal in welke mate huisartsen een telefonisch spreekuur moeten houden op niet-spreekuurwerkdagen om het praktijkdoel te bereiken.
- ☐ Leg vast wanneer u dit praktijkdoel bereikt wilt hebben.
- ☐ Stem met uw collega-huisartsen af op welke niet-spreekuurdagen, bij voorkeur vaste dagen in de week, zij een telefonisch spreekuur houden.
    - Houd er rekening mee dat meer tijd voor patiëntenzorg betekent dat er minder tijd overblijft voor andere taken.
- ☐ Bepaal wie, wanneer de nieuwe telefonische spreekuren invoert.
- ☐ Instrueer de assistenten om alleen eigen patiënten van de huisarts in te plannen op dit spreekuur.
- ☐ Besluit hoe en wanneer u de uitkomst van deze verbetersuggestie wilt monitoren.
  Bijvoorbeeld:
    - *Registreer het percentage werkdagen zonder vast spreekuur met een nieuw ingevoerd telefonisch spreekuur per huisarts.*
    - *Houd een overzichtelijk Excel-bestand bij met de namen van de huisartsen en hun bereikbaarheid voor patiëntenzorg op werkdagen zonder vast spreekuur.*

Groot L, *et al. BMJ Open* 2024; 14:e078169. doi: 10.1136/bmjopen-2023-078169

Praktijkdoel 23: Er wordt gewerkt met een telefonisch terugbelspreekuur

In plaats van een telefonisch spreekuur waarbij patiënten bellen en in de wachtrij worden gezet, wordt gewerkt met een inschrijflijst voor het telefonisch spreekuur. Patiënten kunnen zich (laten) inschrijven op deze lijst voor een bepaald tijdstip. Indien mogelijk worden patiënten op dit tijdstip teruggebeld, wat de bereikbaarheid van de huisarts bevordert. Het is hierdoor gemakkelijker voor patiënten om persoonlijk contact te hebben met hun eigen huisarts.

Voorgestelde aanpak en activiteiten:

- ☐ Stel vast welke medewerkers u wilt betrekken bij het praktijkdoel. Registreer en communiceer dit naar de betrokken medewerkers.
- ☐ Bedenk hoeveel patiënten per uur kunnen worden gebeld.
- ☐ Bepaal het tijdsvenster waarin patiënten teruggebeld worden.
- ☐ Bepaal hoe patiënten zich kunnen inschrijven voor het terugbelspreekuur en bedenk hoe dit gecommuniceerd wordt naar hen.
    - *Wilt u dat patiënten zich online kunnen inschrijven voor het telefonisch spreekuur? Neem dan contact op met de softwareleverancier van uw patiëntenportaal.*
- ☐ Bedenk hoe de terugbelspreekuren genoteerd worden in het HIS.
- ☐ Leg vast wanneer u het terugbelspreekuur invoert.
- ☐ Instrueer de assistenten om met de terugbellijst te werken.
- ☐ Besluit hoe en wanneer u de uitkomst van deze verbetersuggestie wilt monitoren.
    - *Bijvoorbeeld: is er een lijst waarop patiënten zich kunnen inschrijven voor het terugbelspreekuur? Ja/nee*

Praktijkdoel 24: De standaardconsultduur is 15 minuten

Uit pilotstudies is gebleken dat een langer consult (vier patiënten per uur) leidt tot minder verwijzingen en minder ziekenhuisopnames dan een korter consult. Meer tijd voor de patiënt zorgt voor rust in de spreekkamer, meer patiënttevredenheid en een betere arts-patiëntrelatie. Dit komt de persoonlijke continuïteit ten goede.

Voorgestelde aanpak en activiteiten:

- ☐ Stel vast welke medewerkers u wilt betrekken bij het praktijkdoel. Registreer en communiceer dit naar de betrokken medewerkers.
- ☐ Neem kennis van de resultaten van pilots naar een langere consultduur en rapporteer uw bevindingen aan uw huisartsenteam.
- ☐ Benoem de organisatorische gevolgen van een langere standaardconsultduur.
    - *Bepaal of het werken met een langere consultduur gevolgen heeft voor andere afspraken omtrent consulten, bijvoorbeeld patiënten mogen nu twee problemen bespreken in plaats van slechts één.*
    - *Assistenten kunnen meer druk ervaren bij het inplannen van afspraken doordat er minder consulten per huisarts-fte beschikbaar zijn.*
    - *Overweeg om taken of consulten te delegeren naar de praktijkondersteuner of een assistent om huisartsen beschikbaarder te maken voor (complexe) patiëntenzorg.*
- ☐ Overleg met uw huisartsenteam hoe u dit praktijkdoel financieel haalbaar maakt.
    - *Overweeg om taken of consulten te delegeren naar de praktijkondersteuner of een assistent om een hoger aantal declarabele consulten te behouden.*
    - *Overweeg om met uw gecontracteerde zorgverzekeraar(s) of zorggroep, te overleggen voor een pilotvergoeding (zie de resultaten van pilots voor voorbeelden).*
- ☐ Overleg met uw huisartsenteam hoe en wanneer u de verlengde standaardconsultduur doorvoert in uw praktijk.

    Bijvoorbeeld:
    - *voer de langere standaardconsulduur stapsgewijs in per huisarts of direct voor alle huisartsen tegelijk.*
    - *Voer de langere standaardconsultduur selectief of gedeeltelijk in tijdens vaste momenten van de dag of bij specifieke doelgroepen (alleen 65+ patiënten)*
- ☐ Bepaal hoe u naar de patiënten communiceert dat de praktijk werkt met een langere standaardconsultduur.
    - *Benadruk zowel de voordelen van een langere consulttijd (meer tijd voor de patiënt, minder doorverwijzingen, meer tevredenheid) als de nadelen (soms langere wachttijd voor een afspraak).*
- ☐ Besluit hoe en wanneer u de uitkomst van deze verbetersuggestie wilt monitoren.
    - *Bijvoorbeeld: is de standaardconsultduur 15 minuten? Ja/nee*

Praktijkdoel 25: Er zijn duidelijke afspraken tussen huisartsen over de bereikbaarheid buiten kantoortijden voor vragen van collega's over patiënten

De huisartsen maken afspraken over intercollegiale bereikbaarheid buiten kantoortijden. Dit betreft niet alleen de bereikbaarheid voor de huisartsenpost, maar ook die voor de eigen huisartsenpraktijk op dagen dat de betreffende huisarts niet aanwezig is. Hierdoor is de huisarts beter bereikbaar voor vragen over zijn of haar patiënten, wat de persoonlijke continuïteit vergroot.

Voorgestelde aanpak en activiteiten:

- ☐ Stel vast welke medewerkers u wilt betrekken bij het praktijkdoel. Registreer en communiceer dit naar de betrokken medewerkers.
- ☐ Leg vast wanneer u dit praktijkdoel bereikt wilt hebben.
- ☐ Stem met uw collega-huisartsen af welke huisartsen, hoe, wanneer en waarvoor bereikbaar zijn bij vragen over eigen patiënten vanuit de huisartsenpost. Hierbij maakt iedere huisarts afzonderlijk duidelijk hoe en wanneer hij of zij of zij te bereiken is buiten kantoortijden.
- ☐ Bepaal wie, hoe en wanneer dit communiceert naar de huisartsenpost, de andere medewerkers van de eigen huisartsenpraktijk en eventuele waarnemers in de praktijk.
- ☐ Bepaal wanneer u dit praktijkdoel bereikt wilt hebben.
- ☐ Besluit hoe en wanneer u de uitkomst van deze verbetersuggestie wilt monitoren.
    - *Bijvoorbeeld: houd een overzichtelijk bestand bij met de namen van de huisartsen en de wijze waarop en wanneer zij buiten kantoortijden bereikbaar zijn bij vragen over patiënten buiten kantoortijden.*

Praktijkdoel 26: **Huisartsen zijn buiten kantoortijden bereikbaar voor terminaal-palliatieve patiënten**

De huisartsen maken afspraken over hun bereikbaarheid buiten kantoortijden voor terminaal-palliatieve patiënten. Dit kan rechtstreeks arts-patiëntcontact zijn, maar kan ook lopen via collega's of de huisartsenpost. Hierdoor is de huisarts beter bereikbaar voor de groep patiënten die daar de meeste behoefte aan heeft (zie onderzoek van Schers et al.).

Voorgestelde aanpak en activiteiten:

- ☐ Stel vast welke medewerkers u wilt betrekken bij het praktijkdoel. Registreer en communiceer dit naar de betrokken medewerkers.
- ☐ Leg vast wanneer u dit praktijkdoel bereikt wilt hebben.
- ☐ Stem met uw collega-huisartsen af welke huisartsen, hoe, wanneer en voor welke vragen bereikbaar zijn voor hun terminaal-palliatieve patiënten buiten kantoortijden.
    - *Werkt u met duodokters of is er een tweede vast aanspreekpunt voor een patiënt? Denk er dan aan om deze tweede huisarts ook te betrekken bij het maken van afspraken over de bereikbaarheid buiten kantoortijden.*
- ☐ Bepaal wie dit op welke manier communiceert naar de huisartsenpost, de andere medewerkers van de eigen huisartsenpraktijk en eventuele waarnemers in de praktijk.
- ☐ Besluit hoe en wanneer u de uitkomst van deze verbetersuggestie wilt monitoren.
    - *Bijvoorbeeld: houd een bestand bij met de namen van de huisartsen en de wijze waarop en wanneer zij buiten kantoortijden bereikbaar zijn voor terminaal-palliatieve patiënten.*

Praktijkdoel 27: Iedere vaste huisarts houdt minstens 3 dagen per week spreekuur

Iedere vaste huisarts is op 3 verschillende dagen per week, minstens een dagdeel beschikbaar voor directe patiëntenzorg. Dit bevordert de bereikbaarheid van de huisarts. Hierdoor is het voor patiënten gemakkelijker om persoonlijk contact te hebben met hun eigen huisarts.

Voorgestelde aanpak en activiteiten:

- ☐ Stel vast welke medewerkers u wilt betrekken bij het praktijkdoel. Registreer en communiceer dit naar de betrokken medewerkers.
- ☐ Leg vast wanneer u dit praktijkdoel bereikt wilt hebben.
- ☐ Maak een of meerdere voorstellen voor een nieuwe verdeling van werkdagen. Houd er rekening mee dat alle huisartsen op minstens 3 verschillende dagen per week, minimaal een dagdeel beschikbaar zijn voor patiëntenzorg.
- ☐ Plan een overleg met uw collega-huisartsen waarin u uw voorstel voor de nieuwe verdeling van werkdagen bespreekt en overlegt hoe kan worden geschoven in de andere verplichtingen en werkuren om het praktijkdoel te bereiken.
- ☐ Bepaal wie, wanneer en hoe de veranderde werkdagen naar de patiënten communiceert.
- ☐ Besluit hoe en wanneer u de uitkomst van deze verbetersuggestie wilt monitoren.

  Bijvoorbeeld:
  - *Registreer het aantal dagen spreekuur per week per huisarts voor en na het behalen van het praktijkdoel.*
  - *Houd een overzichtelijk Excel-bestand bij met de werkdagen van iedere vaste huisarts per week.*

Praktijkdoel 28: Er is minder externe waarneming

Door zo min mogelijk externe waarnemers in te schakelen, zien patiënten óók bij afwezigheid van hun vaste huisarts zo veel mogelijk dezelfde zorgverleners. Dit vergemakkelijkt het opbouwen van een vertrouwensband tussen huisarts en patiënt.

Voorgestelde aanpak en activiteiten:

- ☐ Stel vast welke medewerkers u wilt betrekken bij het praktijkdoel. Registreer en communiceer dit naar de betrokken medewerkers.
- ☐ Leg vast wanneer u dit praktijkdoel bereikt wilt hebben.
- ☐ Bepaal de voorwaarden voor het opvangen van de afwezigheid van een medewerker en bespreek deze voorwaarden met uw collega's.
    - *Bijvoorbeeld: kortdurende afwezigheid van een werknemer (bijvoorbeeld < 2 weken) wordt opgevangen door de eigen medewerkers c.q. alleen bij langdurende afwezigheid van een werknemer wordt een externe waarnemer ingeschakeld.*
    - *Houd er rekening mee dat de werkdruk voor de andere medewerkers kan toenemen.*
    - *Zorg voor een adequate overwerkvergoeding.*
- ☐ Besluit hoe en wanneer u de uitkomst van deze verbetersuggestie wilt monitoren.
  Bijvoorbeeld:
    - *Houd een overzichtelijk bestand bij met de afwezigheid van medewerkers in het afgelopen jaar en de afgesproken waarnemingsoplossing.*
    - *Maak een overzicht van het aantal ingezette waarnemers voor en na deze interventie.*

Praktijkdoel 29: Huisartsen die de zorg voor een patiëntenpopulatie delen, gaan niet gelijktijdig op vakantie

Wanneer de vakanties van huisartsen die een patiëntenpopulatie delen elkaar niet overlappen, is er altijd minstens één eigen huisarts aanwezig die de patiënten uit deze populatie kent. Hierdoor neemt de kans op discontinuïteit van zorg af.

Voorgestelde aanpak en activiteiten:

- ☐ Stel vast welke medewerkers u wilt betrekken bij het praktijkdoel. Registreer en communiceer dit naar de betrokken medewerkers.
- ☐ Leg vast wanneer u dit praktijkdoel bereikt wilt hebben.
- ☐ Bespreek tijdig de vakantieplanning met uw collega-huisartsen. Bepaal hierbij hoe de planning zo aangepast kan worden dat huisartsen die een patiëntenpopulatie delen niet gelijktijdig op vakantie gaan. Streef ernaar dat altijd minimaal één huisarts werkt die bekend is met de patiëntenpopulatie.
    - *In grotere praktijken of in praktijken waar veel huisartsen met jonge gezinnen werken, kan dit praktijkdoel organisatorisch uitdagend zijn.*
- ☐ Besluit hoe en wanneer u de uitkomst van deze verbetersuggestie wilt monitoren.
    - *Bijvoorbeeld: houd een overzicht bij van de vakanties en geplande afwezigheid van alle huisartsen.*

Praktijkdoel 30: Waarnemend huisartsen schrijven standaard een waarnemingsverslag

Na elke waarneming wordt de waarnemer gevraagd een korte schriftelijke overdracht te schrijven: het waarnemingsverslag. De vaste huisarts wordt zo systematisch geïnformeerd over belangrijke gebeurtenissen binnen zijn of haar patiëntenpopulatie. Zo kan hij of zij proactief handelen richting de patiënt en de persoonlijke continuïteit garanderen.

Voorgestelde aanpak en activiteiten:

- ☐ Stel vast welke medewerkers u wilt betrekken bij het praktijkdoel. Registreer en communiceer dit naar de betrokken medewerkers.
- ☐ Bepaal wanneer u het bereikt heeft dat waarnemend huisartsen standaard een warnemingsverslag schrijven.
- ☐ Leg vast wanneer u dit praktijkdoel bereikt wilt hebben.
- ☐ Bepaal, eventueel in overleg, welke zaken in het waarnemingsverslag moeten staan (bijvoorbeeld spoedgevallen, to do's of 'levensgebeurtenissen) en hoe het verslag opgeslagen en overgedragen wordt.
    - *Bij een drukke waarneming kan het veel tijd kosten een overdrachtsdocument te schrijven. Geef daarom een duidelijke instructie over wat minimaal in het waarnemingsverslag moet staan.*
    - *Indien gewenst, kan worden overwogen om onderdelen mondeling over te dragen.*
- ☐ Bepaal wanneer en op welke wijze de waarnemer de instructie ontvangt om het waarnemingsverslag te schrijven.
- ☐ Besluit hoe en wanneer u de uitkomst van deze verbetersuggestie wilt monitoren.
  Bijvoorbeeld:
    - *Staat er een map op de harde schijf van de praktijkcomputer waarin de waarnemingsverslagen worden opgeslagen? Ja/nee*
    - *Registreer het percentage waarnemingen waarvoor de waarnemer een waarnemingsverslag heeft geschreven.*

Praktijkdoel 31: Overwegingen en reflecties van huisartsen zijn systematisch genoteerd in het HIS

Wanneer een huisarts zijn of haar overwegingen op het gebied van diagnostiek of beleid bij een consult noteert, is het voor de huisarts zelf én voor een waarnemer gemakkelijk om doortastend op te treden tijdens een vervolgconsult. Deze vorm van informatiecontinuïteit compenseert voor de gevolgen van persoonlijke discontinuïteit.

Voorgestelde aanpak en activiteiten:

- ☐ Stel vast welke medewerkers u wilt betrekken bij het praktijkdoel. Registreer en communiceer dit naar de betrokken medewerkers.
- ☐ Leg vast wanneer u dit praktijkdoel bereikt wilt hebben.
- ☐ Bepaal op welk moment u dit praktijkdoel bereikt wilt hebben.
- ☐ Overleg met uw collega-huisartsen hoe u overwegingen en reflecties wilt noteren in het HIS en op welke wijze.
    - *Bijvoorbeeld: noteer op de P-regel overwegingen, met behulp van als-dan-situaties ('Als A blijft bestaan, denk dan aan B of doe C'), en gevoelens over het consult ('Ik heb deze patiënt niet gerust weten te stellen').*
    - *U kunt met uw collega-huisartsen bespreken om alleen voor bepaalde patiënten of consulten de overwegingen en reflecties te noteren. Dit bespaart tijd.*
    - *Houd er bij het noteren van reflecties en overwegingen rekening mee dat patiënten (in)direct inzage in hun dossier kunnen hebben en dus kunnen 'meelezen' met wat huisartsen noteren.*
- ☐ Besluit hoe en wanneer u de uitkomst van deze verbetersuggestie wilt monitoren
  Bijvoorbeeld:
    - *Selecteer consulten waarvoor de overwegingen en reflecties zijn genoteerd en evalueer samen met uw collega-huisartsen hoe correct en volledig deze overwegingen en reflecties zijn.*
    - *Registreer periodiek en steekproefsgewijs het percentage SOEP-registraties met genoteerde overwegingen en reflecties.*

Praktijkdoel 32: Praktijkondersteuners en huisartsen informeren elkaar over levensgebeurtenissen van patiënten

Wanneer de praktijkondersteuner van een patiënt verneemt dat hij of zij een levensgebeurtenis heeft meegemaakt, licht de praktijkondersteuner de vaste huisarts van die patiënt in, en andersom.

Voorgestelde aanpak en activiteiten:

- ☐ Stel vast welke medewerkers u wilt betrekken bij het praktijkdoel. Registreer en communiceer dit naar de betrokken medewerkers.
- ☐ Bepaal in welke mate huisartsen en praktijkondersteuners elkaar moeten informeren over levensgebeurtenissen van patiënten om het praktijkdoel te bereiken.
- ☐ Leg vast wanneer u dit praktijkdoel bereikt wilt hebben.
- ☐ Bespreek met uw huisartsenteam en praktijkondersteuners bij welke levensgebeurtenissen de praktijkondersteuners de vaste huisarts van een patiënt moeten inlichten, en andersom.
  - *U kunt bijvoorbeeld een lijst opstellen met gebeurtenissen waarbij de ander altijd ingelicht wordt.*
- ☐ Bepaal wie, wanneer contact opneemt met de patiënt bij een levensgebeurtenis of hoe dit per individuele patiënt afgestemd kan worden.
- ☐ Bedenk hoe u ervoor kunt zorgen dat huisartsen en praktijkondersteuners (blijven) handelen conform de gemaakte afspraken.
  - *U kunt bijvoorbeeld de instructies periodiek herhalen.*
- ☐ Besluit hoe en wanneer u de uitkomst van deze verbetersuggestie wilt monitoren.
  Bijvoorbeeld:
  - *Is er een lijst met levensgebeurtenissen van patiënten? Ja/nee*
  - *Zijn de praktijkondersteuners geïnstrueerd over het informeren van de vaste huisarts bij levensgebeurtenissen? Ja/nee*
  - *Registreren van het aantal levensgebeurtenissen die wel/niet doorgegeven zijn.*

Praktijkdoel 33: De vaste huisarts van een patiënt met diabetes doet standaard één kwartaalcontrole per jaar

Door eigen patiënten die vooral onder behandeling zijn van de praktijkondersteuner zijn zelf periodiek te zien, blijft de huisarts persoonlijk contact houden met zijn of haar eigen patiënten.

Voorgestelde aanpak en activiteiten:

- ☐ Stel vast welke medewerkers u wilt betrekken bij het praktijkdoel. Registreer en communiceer dit naar de betrokken medewerkers.
- ☐ Leg vast wanneer u dit praktijkdoel bereikt wilt hebben.
- ☐ Bepaal welke patiënten met diabetes onder controle zijn van de praktijkondersteuner(s).
- ☐ Bepaal voor welke patiënten u een kwartaalcontrole door de vaste huisarts laat uitvoeren.
    - *Overweeg om de praktijkondersteuner(s) een selectie van patiënten te laten maken om tijd te besparen, zoals patiënten met multimorbiditeit of zorgmijdend gedrag.*
- ☐ Bepaal wie, wanneer de kwartaalcontroles inplant voor deze patiëntengroep en hoe dit gecommuniceerd wordt naar de patiënten.
- ☐ Bepaal hoe u de uitkomst van deze verbetersuggestie wilt monitoren en wanneer u dit wilt doen.
    - *Bijvoorbeeld: registreer het aantal kwartaalcontroles van diabetes per patiënt door de vaste huisarts per jaar.*

Groot L, *et al. BMJ Open* 2024; 14:e078169. doi: 10.1136/bmjopen-2023-078169

Praktijkdoel 34: In het dossier van patiënten met complexe problemen staat een medische samenvatting

In het dossier van alle complexe patiënten staat een samenvatting met relevante medische gegevens voor een soepele overdracht. U mag zelf bepalen wat een 'complexe patiënt' is. Denk bijvoorbeeld aan 65+-patiënten met meer dan twee chronische aandoeningen.

Voorgestelde aanpak en activiteiten:

- ☐ Stel vast welke medewerkers u wilt betrekken bij het praktijkdoel. Registreer en communiceer dit naar de betrokken medewerkers.
- ☐ Bepaal in welke mate de dossier van complexe patiënten een samenvatting moeten bevatten om het praktijkdoel te bereiken.
- ☐ Bepaal, eventueel in overleg, de definitie van een 'complex probleem'.
    - *Complexiteit wordt bepaald door diverse factoren. Denk bijvoorbeeld aan chronische psychische klachten, multimorbiditeit, een nadelige sociale situatie, zorgmijdend gedrag of een combinatie van deze factoren.*
    - *U kunt bepaalde ICPC-codes, of combinaties van meerdere ICPC-codes, gebruiken om patiënten met complexe problemen te identificeren.*
- ☐ Bepaal wie, wanneer en hoe de patiënten met complexe problemen identificeert in het HIS.
- ☐ Overleg welke informatie de samenvatting moet bevatten. Denk bijvoorbeeld aan actuele ziekte-episodes, hoofddiagnosen, medicatiegebruik, 'advance care planning' en contactgegevens van familie of naasten.
    - *Waak voor 'registratiemoeheid' bij zorgverleners.*
- ☐ Bepaal waar in het HIS de samenvatting komt te staan, zodat deze eenvoudig voor iedereen te vinden is.
    - *Weet u niet waar u in het HIS de samenvatting kunt neerzetten? Neem dan contact op met de helpdesk van uw HIS.*
- ☐ Bepaal op welk moment u dit praktijkdoel bereikt wilt hebben.
    - *Houd er bij de planning rekening mee dat een samenvatting maken tijd vraagt, die ten koste kan gaan van patiëntgebonden taken.*
- ☐ Besluit hoe en wanneer u de uitkomst van deze verbetersuggestie wilt monitoren.
    - *Bijvoorbeeld: registreer het percentage patiënten met complexe problemen voor wie een samenvatting in het HIS staat.*
