# Supplementary material for: Optimising personal continuity for older patients in general practice: a cluster randomised stepped wedge pragmatic trial
Source: BMJ Open. 2024 May 21;14(5):e078169. doi: 10.1136/bmjopen-2023-078169 (PMC11110588; doi:10.1136/bmjopen-2023-078169)
Supplement: Supplementary data [file bmjopen-2023-078169supp003.pdf]

**Supplementary material S3**. Pilot study Dutch GPs' views on improving personal continuity.

In August 2017, 20 GPs in Amsterdam were asked during a sessional meeting to provide recommendations on how to optimise personal continuity for older patients in general practice. Their recommendations are described below:

• To form GP couples and discourage patients to see more than two GPs.

• To use the electronic medical record to identify patients seeing more than two GPs, to evaluate the respective quality of care, and to improve care – if necessary.

• To add diagnostic/therapeutic considerations ('if complaint A persists, think of diagnostic B or therapeutic C') and reflections on consultations ('I was not able to reassure this patient') in the electronic medical record of a patient.

• To have weekly GP meeting during which complex older patients with multiple chronic conditions are discussed.

• To create a pop-up screen for complex older patients that provides key diagnoses, medication, follow-up information,

preferences for resuscitation and intubation, and contact information of relatives and other care providers. Out-of-hours services will automatically receive a copy of this pop-up screen.

• To add a "continuity-of-care weight" to the International Classification of Primary Care (ICPC) codes (i.e. how important is continuity of care for this medical diagnosis?), and to use these weights to create a hierarchy in the medical history of a patient.

• Clear-cut appointments between GPs on reachability for out-of-ours care.

• 24x7 telephonic availability of the GP for patients needing palliative and terminal care.

• To educate GP receptionists about the importance of continuity of care (instead of fast access).

• GPs should have consulting hours during at least three different days a week.

• The routine use of search strategies in the electronic medical record for identification of complex comorbid older patients needing continuity of care.

• Repeat prescriptions only by the own GP.

• During consultations, GPs should register a descriptive diagnosis and a differential diagnosis in the electronic medical record of a patient.
