# Supplementary material for: Optimising personal continuity for older patients in general practice: a cluster randomised stepped wedge pragmatic trial
Source: BMJ Open. 2024 May 21;14(5):e078169. doi: 10.1136/bmjopen-2023-078169 (PMC11110588; doi:10.1136/bmjopen-2023-078169)
Supplement: Supplementary data [file bmjopen-2023-078169supp004.pdf]

Supplemental table S1: Primary outcome confounder analysis. Relevant covariates where added one by one to the model. Confounding was defined as a >10% shift of the effect from the main model.

**Personal continuity: GP knows me**					
	Mean difference Follow-up vs baseline	*95% CI lower*	*95% CI upper*	*P-value*	*Effect shift*
Main model[a]	0.049	-0.052	0.151	0.339	
Practice area (rural v.s. urban)	0.046	-0.055	0.147	0.37	6%
Number of GPs	0.052	-0.049	0.153	0.315	-6%
Number of practice assistants	0.05	-0.52	0.151	0.337	-2%
Number of practice nurses	0.05	-0.052	0.151	0.336	-2%
All covariates	0.049	-0.052	0.151	0.339	0%
**Personal continuity: GP shows commitment**					
	Mean difference Follow-up vs baseline	*95% CI lower*	*95% CI upper*	*P-value*	*Effect shift*
Main model[a]	0.032	-0.75	0.139	0.562	
**Practice area (rural v.s. urban)**	**0.027**	**-0.08**	**0.134**	**0.488**	**16%**
Number of GPs	0.033	-0.074	0.14	0.542	-3%
Number of practice assistants	0.031	-0.076	0.138	0.565	3%
Number of practice nurses	0.032	-0.075	0.139	0.56	0%
All covariates	0.029	-0.078	0.136	0.591	9%
**Team continuity/transmural continuity**					
	Mean difference Follow-up vs baseline	*95% CI lower*	*95% CI upper*	*P-value*	*Effect shift*
Main model[a]	0.011	-0.056	0.079	0.741	
Practice area (rural v.s. urban)	0.011	-0.057	0.078	0.754	0%
Number of GPs	0.012	-0.056	0.08	0.73	-9%
Number of practice assistants	0.011	-0.057	0.079	0.749	0%
Number of practice nurses	0.011	-0.056	0.079	0.746	0%
**All covariates**	**0.013**	**-0.055**	**0.08**	**0.716**	**-18%**

GP= general practitioner

All subscales are measured on a 5 point-Likert scale (1: very low. 5: very high).

[a] Adjusted for cluster, exposure to TOOL-kit, baseline differences

Supplemental table S2. Selected strategies and status of implementation during follow-up

	Strategy	Selected from TOOL-kit	Actively implementing strategy during follow-up	
			Month 3	Month 18
1	Every patient is on the personal list of a GP	3	3	3
2	All GPs in the practice have patients registered on their personal list	1	0	0
3	Patient records in the EPR system display a pop-up message with the name of the patient's regular GP	4	4	3
4	Practice assistants should preferably schedule appointments with the patient's regular GP	13	12	12
5	Practice assistants should preferably schedule appointments for patients with complex problems with the patient's regular GP	5	5	5
6	Follow-up telephone consultations for one illness episode are conducted by the patient's regular GP	4	4	3*
7	One problem, one GP	5	5	4*
8	Home visits for housebound patients are conducted by one or two regular GPs	2	2	2
9	Repeat prescriptions are preferably issued by the patient's regular GP	2	2	2
10	Laboratory results are assessed and discussed with the patient by the requesting GP	4	4	3*
11	Part-time GPs work together in duos	3	2	2
12	Patients with complex problems have two regular GPs	9	9	9
13	Part-time GPs working in duos have regular consultation meetings	3	3	2*
14	All GPs in the practice offer face-to-face meetings to newly registered patients aged 65 years and over	7	7	6
15	All GPs in the practice have regular telephone contact with patients with complex problems	6	6	6
16	The patient's regular GP contacts patients when they return home after a hospital admission	8	8	6**
17	Structured and regular identification of patients with low continuity of care	11	10	7*
18	Patients are regularly informed of organisational changes in the practice via the practice website	4	3	3
19	Patients are regularly informed of organisational changes in the practice via a newsletter	7	7	4
20	Patients can request e-consultations	4	4	4
21	Patients can make appointments online with the healthcare provider of their choice	4	4	4
22	GPs offer telephone consultation to their own patients on non-consultation days	1	1	0*
23	A call-back list for telephone consultations is managed by the practice	2	2	2
24	The standard consultation time is 15 minutes	10	9	7*
25	GPs are available outside of office hours for inter-collegial consultations on complex patients	3	3	2
26	GPs are available for terminally ill patients outside of office hours	4	3	3
27	Every permanent GP offers consultation hours at least 3 days a week	0	0	0
28	Minimise the number of locums	1	1	1
29	GPs who share the care of a patient population do not go on holiday at the same time	4	3	3
30	Locum GPs write a handover report	9	9	6*
31	GPs record their medical considerations and personal reflections systematically in the EPR system	6	6	3*
32	Practice nurses and GPs inform each other of patients' life events	3	3	3
33	The patient's regular GP performs one quarterly diabetes check-up per year in their diabetic patients by default	4	4	3
34	The records of patients with complex problems contain a medical summary	8	7	5*

GP= general practitioner, EPR= electronic patient register
* one practice withdrew from the trial
** two practices withdrew from the trial

Supplemental table S3. Acceptability outcomes of the preliminary process evaluation

Outcome		Baseline	Start intervention	18-month Follow-up
		n=161	*n=180*	*n=121*
			N (%)	
Ability to improve personal continuity[1]	(very) high	91 (57)	105 (59)	69 (52)
	Moderate	63 (39)	64 (35)	45 (34)
	(very) low	7 (4)	10 (6)	6 (5)
	*Non-response**	*0 (0)*	*0 (0)*	*12 (9)**
Priority to improve personal continuity[2]	(very) high	145 (90)	156 (87)	93 (71)
	moderate	15 (9)	16 (9)	24 (18)
	(very) low	1 (1)	6 (3)	4 (3)
	*Non-response**	*0 (0)*	*1 (1)**	*11 (8)**

* not all participants completed the survey
1 Measured on a five-point Likert scale: 1 = very low, 5= very high)
2 Measured on a 10-point scale: (1= very low, 10= very high)

Supplemental table S4. Process evaluation outcomes: estimated measurements of all strategies of TOOL-kit.

Strategy	Outcome	Estimated measurement during Follow-up				Steps completed from practice improvement plan*		
		3 month		18 month		18-month		
		Measure (mean)	*Response*	Measure (mean)	*Response*	*All*	*Partially*	*None*
1. Every patient is on the personal list of a GP	Percentage of patients who have been registered on the personal list of a GP	50%	*2/3*	0%	*1/2*	0	2	0
2. All permanent GPs in your practice have patients registered on their personal list†	Percentage of permanent GPs with a personal list	n.a.†		n.a.†		n.a.†		
3. Patient records in the EPR system display a pop-up message with the name of the patient's regular GP	Percentage of patient records that have a pop-up message showing the name of the patient's regular GP	33%	3/4	20%	2/3	1	2	0
4. Practice assistants should preferably schedule appointments with the patient's regular GP	Percentage of consultations scheduled with regular GP of a patient	64%	*6/12*	74%	*9/12*	1	8	3
5. Practice assistants should preferably schedule appointments for patients with complex problems with the patient's regular GP	Percentage of consultations scheduled with the own GP of a complex patient	88%	*2/5*	84%	*2/5*	1	2	0
6. Follow-up telephone consultations for one illness episode are conducted by the patient's regular GP	Percentage of follow-up telephonic consultations scheduled with the own GP of a patient	83%	*2/4*	75%	*2/3*	1	1	0
7. One problem, one GP	Percentage of follow-up consultations scheduled with the GP that had first seen a patient	73%	*4/5*	80%	*2/4*	0	2	1

8. Home visits for housebound patients are conducted by one or two regular GPs	percentage of home visits to house-bound patients done by the regular GP of that patient	60%	*1/2*	75%	*2/2*	0	2	0
9. Repeat prescriptions are preferably issued by the patient's regular GP	Percentage of repeat prescriptions issued by the patient's regular GP	80%	*1/2*	70%	*2/2*	0	1	1
10. Laboratory results are assessed and discussed with the patient by the requesting GP	Percentage of laboratory results dealt with by the patient's regular GP	85%	*2/4*	NR	*0/3*	0	3	0
11. Part-time GPs work together in duos	Percentage of permanent GPs working as duo-doctors	50%	*2/2*	50%	*2/2*	1	0	1
12. Patients with complex problems have two regular GPs	Percentage of complex patients with two regular GPs	64%	*5/9*	55%	*6/9*	1	2	2
13. Part-time GPs working in duos have regular consultation meetings	the number of consultation meetings between duo-doctors per month	NR	*0/3*	0	*1/2*	0	1	0
14. All GPs in the practice offers a face-to-face meeting to newly registered patients aged 65 years and over	Percentage of new patients aged 65 and over who are offered an introductory interview	58%	*3/7*	38%	*4/6*	3	2	2
15. All GPs in the practice have regular telephone contact with patients with complex problems	Percentage of complex patients who are on a call list of their own GP	65%	*2/4*	53%	*2/4*	0	0	4
	Percentage of GP's who have a call list with their complex patients	63	*1/1*	90%	*1/1*	1	0	0
	Frequency of GP-initiated contact in the past month	Monthly: 1	*1/1*	3-monthly: 1	*1/1*	1	0	0

16. The patient's regular GP contacts patients when they return home after hospital admission for a treatment or event that had a profound impact on the patient	Percentage of patients who were contacted by their regular GP after hospital admission for a major event.	76%	*3/8*	61%	*6/6*	1	2	3
17. Structured and regular Identification of patients with low continuity of care	Usual Provider Continuity index	n.a. ‡		n.a.‡		1	4	1
18. Patients are regularly informed of the current staff members and of working hours and working days of healthcare providers via the practice website	Score on 6-point Likert-scale Checklist on quality of information on the practice website (max: 30 points, average score displayed)	22	*3/3*	24	*1/3*	1	2	0
19. Patients are regularly informed of changes in staff and in working hours and working days of healthcare providers via a newsletter	Number of digital newsletters send in the past 12 months	2	*3/7*	0,33	*3/4*	0	3	1
20. Patients can request e-consultation	Yes/no question 'can patient request an e-consultation with their healthcare provider?	Yes: 0<br>No: 3	*3/4*	Yes: 3<br>No: 0	*3/4*	2	1	1
21. Patients can make appointments online with the healthcare provider of their choice	Yes/no question 'can patients make appointments online?'	Yes: 0<br>No: 3	*3/4*	Yes: 1<br>No: 1	*2/4*	0	2	1
22. GPs offer telephone consultation to their own patients on non-consultation days†	Percentage of non-consultation days on which a time slot has been newly allocated to telephone consultation hours per GP	0	*1/1*	n.a.†			n.a.†	
23. A call-back list for telephone consultations is managed by the practice	Yes/no question 'Is there a call-back list which patients can subscribe to?'	NR	*0/2*	Yes: 1<br>No 0	*1/2*	0	1	0

24. The standard consultation time is 15 minutes	Count of GPs working with 15 minute consultation time	All: 6. Mostly not: 1	*7/9*	All: 4 Most: 1	*5/7*	4	1	0
25. GPs are available outside of office hours for inter-collegial consultations on complex patients	Percentage of GPs with whom their out-of-office availability is discussed	50	*2/3*	75	*2/2*	1	1	0
26. GPs are available for terminally ill patients outside of office hours	Percentage of GPs with whom their out-of-officie availability for terminally ill patients is discussed	50	*1/3*	100	*1/3*	1	1	1
27. Every permanent GP offers consultation hours at least 3 days a week	Count the consultation days per GP per week before and after achieving the practice goal.	n.a.†		n.a.†			n.a.†	
28. Minimalise the number of locum GPs	Count the locum GPs your practice has employed before and after this intervention.	10	*1/1*	10	*1/1*	0	1	0
29. GPs who share the care of a patient population do not go on holiday at the same time	Yes/no question 'Is there overlap between vacation of GPs who share a patient population'	Yes: 2 No: 1	*3/3*	Yes: 1 No: 0	*1/3*	0	2	1
30. Locum GPs write a handover report	Percentage of locum shifts for which the locum GP has written a handover report	0	*4/9*	20	*5/6*	0	2	3
31. GPs record their medical considerations and personal reflections systematically in the EPR system	Percentage of consultations for which considerations and reflections have been recorded in the GP EPR system	25	*2/6*	35	*1/3*	0	1	2
32. Practice nurses and GPs inform each other of patients' life events	Percentage of life events that have/have not been passed on	70	*1/3*	75	*2/3*	0	2	1

33. The patient's regular GP performs one quarterly diabetes check-up per year in their diabetic patients by default	Percentage of quarterly diabetes check-ups performed by the patient's regular GP per patient per year	27	*3/4*	60	*3/3*	1	1	1
34. The records of patients with complex problems contain a medical summary	Percentage of patients with complex problems for whom the patient record contains a summary	2%	*5/7*	0	*1/5*	0	2	2

GP= general practitioner, EPR= Electronic Patient Register, NR= non-response

**Steps completed* was determined by reviewing practice responses from T1 to T6. The last known response of a practice as taken as an end point to determine the extend in which the steps were undertaken. As practices' response rate varied (i.e. a practice could complete T1-T5 but not T6) the number of responses from the 18 month follow-up does not always align with the number of practices in *steps completed.*
† Strategy was not implemented by a practice
‡Strategy was implemented but outcome was not calculated by a practice
